# Insight into the Precipitation Inhibition of Polymers within Cocrystal Formulations in Solution Using Experimental and Molecular Modeling Techniques

**DOI:** 10.1021/acs.cgd.4c01573

**Published:** 2025-02-28

**Authors:** Peace Alinda, Adolfo Botana, Mingzhong Li

**Affiliations:** † Leicester School of Pharmacy, De Montfort University, Leicester LE1 9BH, U.K.; ‡ JEOL (U.K.) LTD., Welwyn Garden City AL7 1LT, U.K.

## Abstract

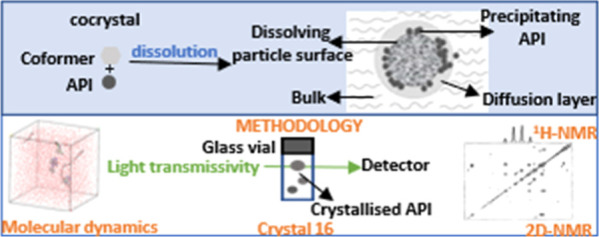

This study investigated the role of various polymers as precipitation inhibitors in solutions of flufenamic acid (FFA) and its cocrystals with theophylline (FFA–TP) and nicotinamide (FFA–NIC). Through a combination of NMR spectroscopy, molecular dynamics simulations, and nucleation studies using Crystal16, we evaluated the effects of polyethylene glycol (PEG), polyvinylpyrrolidone–vinyl acetate (PVP–VA), and soluplus (SOL), both individually and in combinations, on the nucleation, diffusion, and self-association of FFA molecules in solution. ^1^H NMR and DOSY measurements revealed that while PEG was highly effective in reducing molecular mobility, thus significantly delaying nucleation, PVP–VA facilitated nucleation by enhancing FFA diffusion and aggregation. SOL provided a balance, enhancing molecular mobility but maintaining a delayed nucleation effect, likely due to micellar encapsulation, as evidenced by line broadening in ^1^H NMR. Combination systems such as PVP–VA–PEG and PVP–VA–SOL showed synergistic effects, with PVP–VA–SOL proving particularly effective in inhibiting FFA nucleation across all systems. Molecular dynamics simulations supported these findings by highlighting changes in intermolecular interactions and aggregation tendencies in the presence of each polymer. This comprehensive analysis suggested that selecting appropriate polymeric excipients, or combinations thereof, can finely tune the nucleation behaviors of drug solutions, offering a strategic approach to optimizing the stability of supersaturated drug solutions.

## Abstract 2

This study investigates the role of polymers in modulating precipitation of flufenamic acid (FFA) from cocrystal solutions using NMR, molecular dynamics, and nucleation studies. Results reveal how polymer interactions influence dissolution, diffusion, and nucleation, aiding in the rational design of cocrystal formulations to enhance drug stability and bioavailability.

## 1 Introduction

Pharmaceutical cocrystals have become one of the essential formulation strategies for developing solid dosage forms of drug products in the pharmaceutical industry to tackle the formulation challenges posed by low solubility drug compounds.^1−3^ However, translational development of pharmaceutical cocrystals into drug products, such as tablets and capsules, can be challenging due to the safety of the coformers, formulation and manufacturing process design, and unpredictable in vivo performance.^4^ In terms of formulation challenges, it is essential to maintain the supersaturation state (the “spring”) of the parent drugs generated by the rapid dissolution of pharmaceutical cocrystals, often referred to as the “parachute” effects, to achieve the improved bioavailability of the drug products.^5−7^ This requires an optimal combination of excipients, parent drug and cofomer.^8,9^ During dissolution, the parent drug of the cocrystal could be recrystallized on the dissolving surface and/or in the bulk solution, therefore, a polymeric excipient is needed in the formulation, acting as a precipitation inhibitor to prevent the parent drug recrystallization.^10−12^ Furthermore, in a recent study, we have demonstrated that a combination of two polymeric excipient inhibitors can generate a synergistic inhibition effect, further maximizing a cocrystal’s potential.^13^ Currently, the selection of a polymeric inhibitor or a combination within a cocrystal formulation largely relies on an experimental trial-and-error approach. To quickly and reliably determine potential polymeric inhibitors from a large pool of candidates, a comprehensive molecular understanding of the interactions of the components in solution is needed to provide a scientific basis for excipient selection.

Solution nuclear magnetic resonance (NMR) spectroscopy is a powerful tool for investigating and understanding the molecular state of drugs in solution.^14−17^ Solution proton NMR (^1^H NMR) data can provide information about the interactions of molecules where the complexation of different compounds and/or self-association within the same compounds induce changes in their chemical shifts.^17^ However, ^1^H NMR data cannot provide information about the specific interaction site between two molecules. As nuclear overhauser effect spectroscopy (NOESY) shows through-space interactions within the molecule, rather than the through-bond interactions seen in the other methods, NOESY measurements can provide information on identifying the intermolecular interaction sites between different components in solution.^18,19^ In a supersaturated solution, the mobility of the molecules is important, which determines the probability of collision of drug molecules for the formation of nuclei of a critical size, leading to precipitation from solution.^20^^1^H NMR peak width of the molecules reflects their molecular mobility, for example, NMR peak broadening demonstrates mobility suppression.^21,22^ In order to quantify the mobility of the drug molecules, the diffusion coefficient of the molecules needs to be measured. This coefficient is directly related to its molecular size, aggregation, and binding phenomena in solution.^23^ Diffusion-ordered NMR spectroscopy (DOSY) enables the measurement of translational diffusion (conventionally defined as self-diffusion) of dissolved molecules and provides direct information on molecular dynamics, including intermolecular interactions and conformational changes.^24^ DOSY has widely been used to study the complexation of the host and guest molecules, as tightly bound complexes, often exhibit the same diffusion coefficient as a single molecular entity.^25^

Molecular dynamics (MD) simulation is another powerful and promising approach to understanding the interaction of molecules in solution, which is capable of visualizing the real-space arrangement of molecules at length scales inaccessible to experimental techniques.^26−33^ Although the length and time scales of the simulations are not enough to be conclusive, the interactions between the drug molecules and polymer inhibitors can be examined and interpreted from several indictors, including solvent accessible surface area (SASA), radial distribution function (RDF), hydrogen bonds (HBs), and energy analysis. Most studies have found that there were few or no hydrogen bonds formed between drug molecules and a polymeric excipient in solution. Instead, non-hydrogen bonds such as coulomb and Lennard-Jones (LJ) interactions, contribute to maintaining the drug molecules in solution by suppressing drug aggregation and mobility.^29,30^ The MD simulation results were found to be in excellent agreement with the experimental observations, and they can be used to rank excipients’ relative efficiency within a formulation.^33−35^ Currently integrated computer-aided formulation design approach has been widely applied for pharmaceutical product development.^36−38^

In this study, we reported our research on mechanistic understanding of the interactions between the drug molecule flufenamic acid (FFA), coformers [i.e., nicotinamide (NIC) and theophylline (TP)] and polymeric excipients [i.e., polyethylene glycol (PEG), copolymer of vinylpyrrolidone (60%)/vinyl acetate (40%) (PVP–VA), and soluplus (SOL)] within a cocrystal formulation in solution using experimental and molecular modeling techniques. The pharmaceutical cocrystals, i.e., flufenamic acid and theophylline (FFA–TP) and flufenamic acid and nicotinamide (FFA–NIC), have been extensively studied and characterized in our group. These studies have shown that their dissolution behaviors can be regulated by polymeric excipients, such as PEG, PVP–VA, or SOL, or a combination of PVP–VA and SOL in the dissolution media.^10−12,39,40^ Structures of the individual molecules, cocrystals and polymers are shown in Table 1. NMR measurements including ^1^H, DOSY and NOESY were conducted on singular, binary and ternary component systems. These systems included a parent drug (FFA), coformer (TP or NIC) and polymer (PEG, PVP–VA, or SOL) in a cosolvent of 50% deuterated dimethyl sulfoxide (DMSO) and 50% deuterium oxide (D_2_O). The possible interactions between drug/coformer, drug/polymer or coformer/polymer were explored by the NOESY measurements. Changes in the self-association properties of the parent drug molecules of cocrystals, in the absence and presence of a polymer, were assessed using characteristic chemical shifts from the ^1^H NMR data. Quantification of these changes was based on diffusion coefficients determined from the DOSY measurements.

**Table 1:**
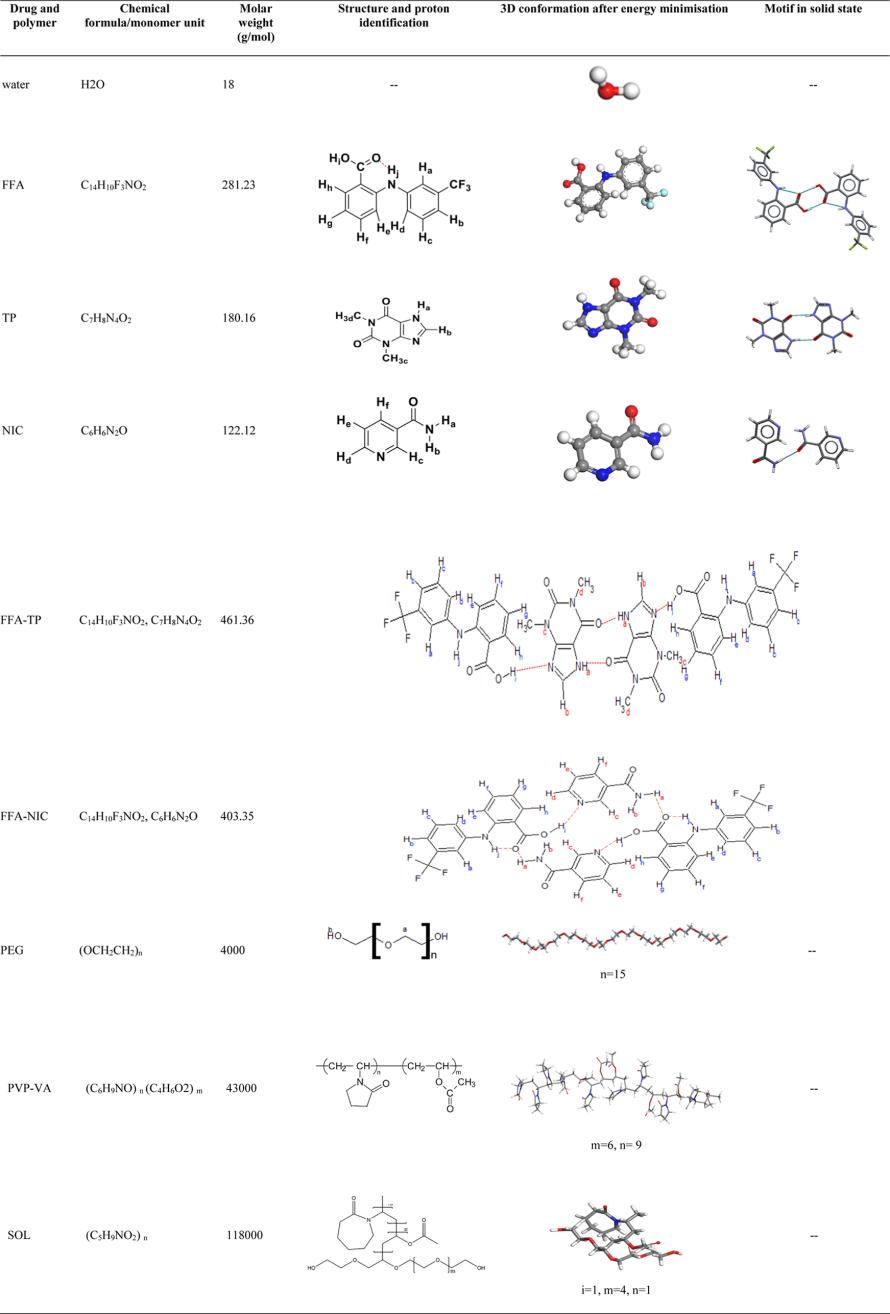
Chemical Structures of Cocrystals and Monomer Units of Polymers

In parallel, the MD simulations of aqueous solutions containing singular (drug molecules), binary (drug/coformer or drug/polymer) or ternary (drug/coformer/polymer) components were conducted using commercial software packages of Biovia Materials Studio (V2020) to mimic the NMR experiments. These simulations provided insights into the experimental phenomena at a molecular level by examining changes in drug molecule interaction energies (e.g., van der Waals forces and electrostatic forces), mean length evolution, mean squared displacement (MSD), and RDF. Finally, the interplay between the drug, coformer and polymeric excipients on the precipitation behaviors of the parent drugs in solution was validated through the nucleation experiments of the different cocrystal formulation systems using Crystal16.^41^

## 2 Materials and Methods

### 2.1 Materials

Flufenamic acid (FFA) (≥97% purity), nicotinamide (NIC) (≥99.5% purity), theophylline (TP) (≥99.5% purity) and Poly(ethylene glycol) (PEG) 4000 were purchased from Sigma-Aldrich (Dorset, U.K.). Plasdone S-630 (PVP–VA, 43,000) was a gift from Ashland Inc. (Schaffhausen, Switzerland). Soluplus (SOL) was a donation by BASF (Ludwigshafen, Germany). Acetonitrile (≥99.8% purity, HPLC grade) and methyl sulfoxide-*d*_6_ (DMSO, 1 v/v %TMS, 99.8 atom %D) were purchased from Fisher Scientific U.K. (Loughborough, U.K.). Double-distilled water was generated from a Bi-Distiller (WSC044.MH3.7, Fistreem International Limited, Loughborough, U.K.). Deuterium oxide (D_2_O, 99.9%) was purchased from Goss Scientific (Crewe, UK).

### 2.2 Methods

#### 2.2.1 Cocrystal Synthesis

The FFA–NIC and FFA–TP cocrystal powders were generated by cooling crystallization using Polar Bear Plus Crystal (Cambridge Reactor Design Ltd., UK). A 1:1 molar ratio mixture of FFA and TP or NIC was added to the cosolvent (70% acetonitrile/30% water) in a 20 mL vial which was held at 45 °C until all solids were dissolved. The temperature was then reduced to 0 °C at a rate of 0.3 °C/min. The powders formed were isolated by paper filtration. Before any further use, the dried powders were analyzed by PXRD to confirm the cocrystal formation. The observed patterns were compared to the predicted patterns retrieved from the Cambridge Structural Database (CSD): FFA–TP (ZIQDUA) and FFA–NIC (EXAQAW).

#### 2.2.2 ^1^H, NOESY and DOSY NMR Measurements

^1^H NMR, diffusion ordered spectroscopy (DOSY) and Nuclear Overhauser effect spectroscopy (NOESY) experiments were performed using the JEOL ECZ 600R series FT-NMR Spectrometer (JEOL Ltd., Tokyo, Japan) at 25 °C using a 5 mm NMR sample tube. Measurements were conducted in a cosolvent of 50% DMSO and 50% D_2_O. The DMSO also contained 1 v/v % tetramethylsilane (TMS) as an internal standard. The FFA concentration in each of the solutions was constant at 0.5 mg/mL while the concentrations of TP and NIC were 0.32 and 0.1 mg/mL, respectively corresponding to the equal molar ratio of FFA cocrystals. To investigate the effect of the different types of polymers and their combination on the cocrystal systems, PVP–VA, SOL, and PEG as well as combinations of PVP–VA–PEG and SOL-PVP–VA were studied at three different concentrations: 0.025, 0.05, and 0.25 mg/mL for PVP–VA and PEG and 0.01, 0.025, and 0.05 mg/mL for SOL. Details of the samples and their preparation can be found in Table S1 in the Supporting Information.

For the ^1^H NMR measurements, a single pulse was used as the excitation pulse with a 5 μs pulse width. The acquisition was performed with a relaxation delay of 2 s between scans, utilizing a phase cycling scheme. A total of 32 scans were collected with an acquisition time of 2.42221 s. The 1D spectral data were processed using the JEOL Delta v5.3.1. software.

The NOESY experiments were run with a phase-sensitive sequence with a mixing time of 1 s. The acquisition was carried out in two scans, each consisting of 4096 (*X*) × 256 (*Y*) complex points, with a total of 512 scans. The relaxation delay was set to 1.5 s, and the repetition time was 1.80278 s. The NOESY data were processed using JEOL Delta v5.3.1. software.

The DOSY experiments were acquired with a convection compensated stimulated echo with bipolar gradient pulse sequence, with 16 equal increments in gradient squared, ranging from 30 to 300 mT/m. The relaxation delay was 5 s and the total acquisition time was 1.4549 s. The spectra were phase and baseline-corrected and analyzed using the General NMR Analysis Toolbox (GNAT) 1.3 2021 software to estimate the diffusion coefficients.

#### 2.2.3 Nucleation Study

A supersaturation level of 1.475 of the pure FFA in the cosolvent of 50% acetonitrile/50% water and in the presence of 0.005 mg/mL of a single polymer (i.e., PEG, SOL) or a combination of two polymers (i.e., PVP–VA–PEG and PVP–VA–SOL) each at 0.005 mg/mL at 27 °C was selected in the study. To compare the performance of the polymers in maintaining FFA in solution, an equimolar concentration of FFA was used to prepare the solutions of FFA–TP and FFA–NIC cocrystals. One milliliter aliquots of the supersaturated solutions, prepared at 45 °C, in the presence or absence of polymers, were transferred to a 2 mL HPLC clear glass vials containing a magnetic stirrer (3× 8 mm) using a plastic pipet. The vials were tightly closed with screw caps to minimize evaporation. After the vials were placed into Crystal16, the reactor temperature was rapidly increased to 45 °C and maintained for 20 min to ensure the complete dissolution of solid particles in the vials. The clear solution was then cooled to 27 °C at a rate of 5 °C/min, which was the maximum cooling rate to ensure that the vials’ temperature followed the desired temperature ramp. The reactor was then held at 27 °C for several hours. The light transmission through the sample decreased when the nucleation of solids started in the vial, which is called the cloud point. The induction time was taken as the difference between the time of the earliest detection of nucleation in the sample vial and the time zero at which the solution temperature in the vial started to decrease below 45 °C at the beginning of each cooling cycle. The time zero selected here was different from those in many other studies where the moment that the solution reached the desired nucleation temperature was the time zero. The proposed new time zero strategy can avoid any negative induction time that was observed during the experiments because crystal nucleation is a stochastic process and can occur at any time during the temperature. More details of the experiments can be found in our previous publication.^41^

#### 2.2.4 Molecular Dynamics Simulations

Simulations were performed using the BIOVIA Materials Studio (2020, v20.1.05) software package. The atomic charges (O = −8476e, H = +0.4238e) for the water molecule were adapted from the extended simple point charge (SPC/E) model.^42^ Charges for other molecules were force field assigned using the Gasteiger method. The cubic box was packed with 3000 water molecules and the other molecules using the amorphous cell construction tool in the Materials Studio. The size of the box generated (approximately 45 Å × 45 Å × 45 Å) was determined by the software based on the number of molecules. Periodic boundary conditions (PBC) were maintained throughout all of the simulations. The density of the simulation box was approximately equal to the bulk density of water, 1 g/cm^3^. The simulation conditions and settings including force field type, simulation time, energy summation method, barostat type and ensemble among other settings were decided by simulating 3000 water molecules alone in a cubic box and adjusting the simulation parameters until the bulk properties of water were close enough to the experimental values, such as the density of 1 g/cm^3^ and diffusion coefficient is 2.3 × 10^–9^ m^2^ s^–1^. Other indicators of system validity like energy, temperature and pressure were also monitored. The trajectories were analyzed and the mean square displacement (MSD) of the water molecules was determined and used to calculate the diffusion coefficient (*D*). The change of MSD with time is related to the diffusion coefficient D by the expression
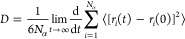
1where *N*_α_ is the number of diffusive atoms in the system and *r* is the distance of the particle *i* at a time *t*. The diffusion coefficient is calculated in units of Å^2^/ps and converted to m^2^/s by dividing the resulting value by 1 × 10^8^. The combination of settings that resulted in a calculated diffusion coefficient of water closest to the experimental value of *D* = 2.3 × 10^–9^ m^2^ s^–1^ and with all other indicators valid was applied to all simulations. The simulation steps are summarized as follows.

#### 2.2.5 Construction of Starting Structures

The molecular structures of FFA, TP and NIC were imported into the Materials Studio from CSD (reference codes: FPAMCA11, BAPLOT01 and NICOAM02). The molecules were automatically assigned charges by the Dreiding force field using the Gasteiger charge method.

The water molecule was sketched using the Materials Studio Visualizer sketching tools and the SPC/E charges were manually assigned to the atoms (H = 0.424, O = −0.847). The polymers were built using the polymer builder tools after defining the head and tail atoms of the repeat unit. For purposes of computational efficiency, one polymer chain consisting of 15 repeat units was used for PEG, representing 17% of the average molecular weight used in the experiments. The PVP–VA was constructed as a random copolymer with 9 repeat units of PVP and 6 repeat units of vinyl acetate, representing 4% of the polymer molecular weight used in the experiments. SOL, which is a tricopolymer of PEG, polyvinyl acetate (PVAc) and polyvinyl caprolactam (PVCL), was represented by 1 monomer unit (4PEG 1VAc 1VCL), representing 0.4% of the polymer molecular weight used in the experiments. The atoms were also automatically assigned charges using the Dreiding force field with the Gasteiger charge method.

**Figure 1:**
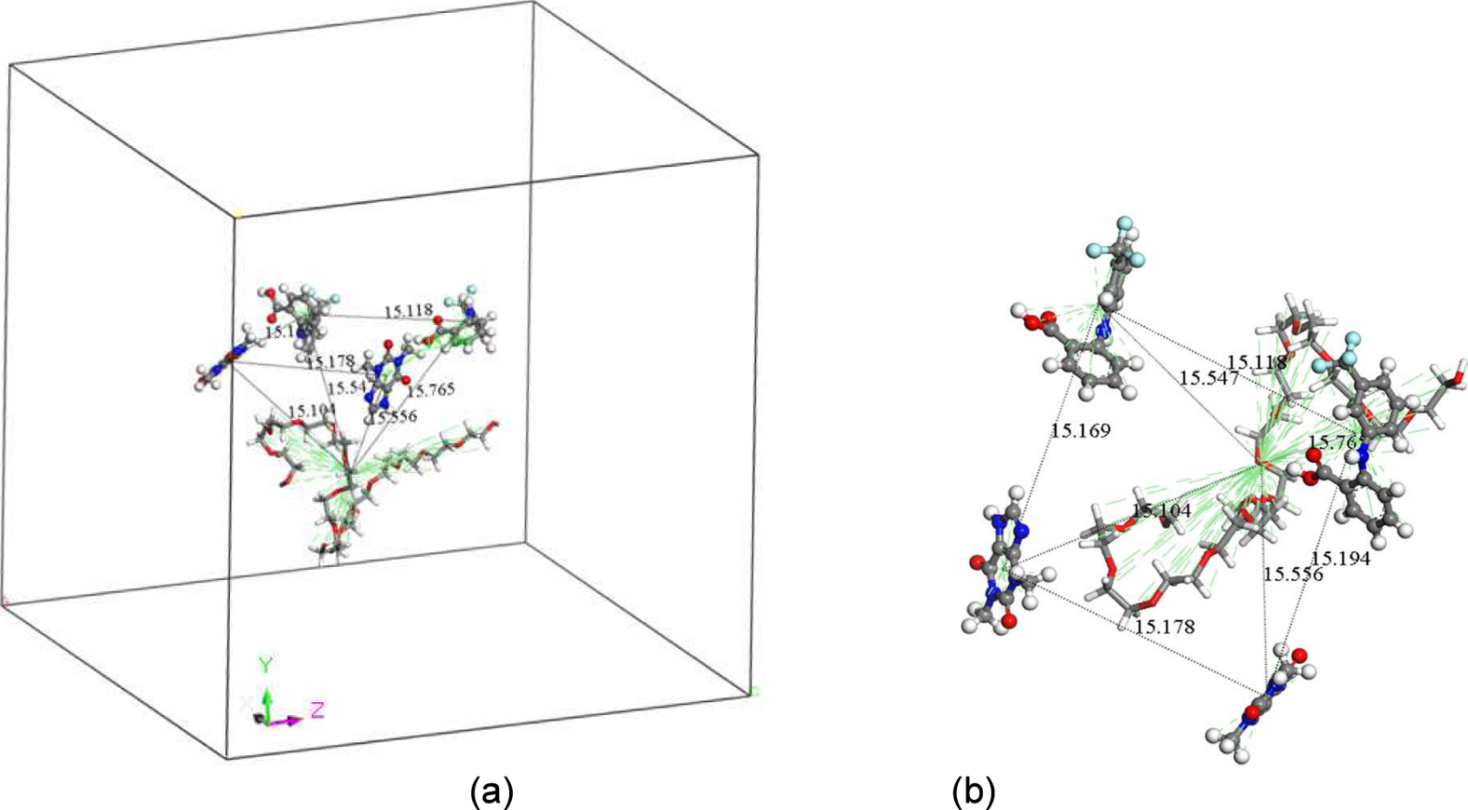
(a) The amorphous cell containing the molecules of FFA and TP in the presence of PEG (b) starting positions between the molecules.

All the molecules were geometry optimized before being added to an amorphous cell. The amorphous cell module was used to define the composition of the amorphous cell and build a cubic water box at the temperature of 300 K and density of 1 g/cm^3^. Either two FFA molecules alone or with two coformer molecules (i.e., TP or NIC) in the absence and presence of a polymer or a combination of two polymers (SOL/PVP–VA or PEG/PVP–VA) were added in the water box. Initially, the molecules had position restraints applied to their centroids allowing the simulations to start with the drug molecules at distances between 9 and 16 Å apart from each other. The position restraints were then removed before running the production dynamics simulations. An example of the amorphous cell containing the molecules of FFA and TP in the presence of PEG and the starting positions between molecules are shown in Figure 1 (water molecules hidden for visibility). Those of the other simulations are shown in Figure S9 in the Supporting Information.

#### 2.2.6 Relaxing the Amorphous Cell

The amorphous cell was subjected to three main steps to achieve a relaxed cell with minimum energy:

a Geometry Optimization: Initially, the molecules within the box were geometry optimized using the smart descent algorithm without optimizing the cell parameters for 2000 iterations. This was followed by a second geometry optimization using the steepest descent algorithm, this time including optimization of the cell parameters.
b Quench Dynamics: The amorphous cell was then subjected to quench dynamics for a total time of 50 ps with a time step of 1 fs under *NPT* ensemble conditions, which ensured that the number of particles, pressure (0 GPa), and temperature (300 K) remain constant. The frame with the minimum converged energy was selected for the third stage.
c Annealing: The selected structure was annealed in 5 cycles, from an initial temperature of 300 K to a midcycle temperature of 500 K, under the *NVE* ensemble (constant number of particles, volume, and energy). Each output structure was optimized, and the structure with the minimum converged energy was chosen to proceed to the dynamics step.

#### 2.2.7 Molecular Dynamics

To attain equilibrium, molecular dynamics simulations were first run for 1050 ps at a constant temperature of 300 K controlled by the Nose–Hoover thermostat, under *NVT* ensemble conditions. The time step was 1 fs. The Dreiding force field was used for all the simulations. Different summation methods were used for different terms: Ewald mesh summation for electrostatic terms, group-based summation for van der Waals (VDW) terms and, atom-based summation for hydrogen bond terms. The cutoff distance was 15.5 Å for van der Waals terms and 4.5 Å for hydrogen bonding. The production run was performed under the *NPT* ensemble for 1100 ps, with frame output at every 10,000 steps. The temperature was controlled by the Nose–Hoover thermostat and the pressure by the Parrinello–Rahman barostat. All other conditions remained the same. Data analysis includes:

a Distance evolution: The evolution of the centroid-to-centroid distance in angstroms (Å) between two molecules in the trajectory was analyzed using the Forcite module in Materials Studio. The distance (Å) was then plotted against time (ps) to visualize the distance evolution between the molecules. In cases where excipient molecules were added, the distance between each FFA molecule and each excipient molecule was measured, and the average distance was calculated. This average distance was then plotted against time, as shown in Figure S9 in the Supporting Information.
b Diffusion coefficient: The MSD of the FFA molecules in each simulation was analyzed using 55 frames produced over 550 ps (half the simulation time) with the Forcite module. The diffusion coefficient was determined from the MSD in Materials Studio using eq 1. The graphs of MSD (Å^2^) of FFA molecules against time (ps) are shown in Figure S10 in the Supporting Information.
c Radial distribution function: RDF was analyzed using the Forcite analysis module in Materials Studio. This analysis generated a plot of *g*(*r*) against radius, which represents the probability of finding a pair of particles at a specific distance from each other compared to a random distribution. The plot reveals the highest peak at a certain radius, indicating the most probable distance between FFA molecules and excipient molecules. This radius highlights the preferred separation distance between these molecules, providing insight into their spatial distribution and interaction within the simulation (Figure S11 in the Supporting Information).
d Nonbond energies: The nonbond energy (Table 3) between the FFA molecules in each simulation was computed from the final frame after the production run. Water and excipient molecules were removed from the water box, and the energy of the FFA molecules alone was computed using the Forcite module. The total nonbond energy includes contributions from hydrogen bonding, VDW forces, long-range corrections, and electrostatic interactions.

## 3 Results

### 3.1 NMR Results

Assignments of the ^1^H chemical shifts of the protons of FFA, coformers (TP and NIC) and polymers were based on the previous work^40,43−45^ shown in Figure S2 in the Supporting Information. The focus of this work was to examine the changes in the characteristic chemical peak shifts of the FFA molecules caused by the self-aggregation of FFA molecules or interaction with the environment in solution. Based on the conformation of the FFA molecule (Table 1), the chemical shifts of two doublet peaks of H_h_ at 7.99 ppm, triplet peaks of H_g_ at 6.98 ppm, and singular peak H_b_ at 7.38 ppm are prone to the change of the environment and intermolecular interaction with the other components in solution while the chemical shifts of characteristic peaks H_e_ at 7.35 ppm and H_a_, H_c_, H_d_ and H_f_ in the range of 7.57–7.47 ppm will mainly be affected by intramolecular interaction of FFA due to change of the molecule conformation.

### 3.2 One-Dimensional ^1^H NMR Spectra Analysis

#### 3.2.1 Effects of Polymers on the Chemical Shifts of FFA in the FFA alone Solution

The one-dimensional ^1^H NMR spectra were analyzed to investigate the effects of polymers on the chemical environment of FFA protons in solution, as well as in the presence of cocrystal coformers (TP and NIC). Changes in the chemical shifts of key FFA protons (H_h_, H_c_, H_b_, H_e_, H_g_) were observed upon polymer addition, reflecting specific interactions between the polymers and the FFA molecules. Overlapping proton peaks (a, c, d, f) were combined under a single peak “H_c_” (tracked by the tallest peak in the multiplet) in the analysis. The chemical shifts for the FFA protons in solutions of FFA, FFA–TP and FFA–NIC, with and without polymers, are presented in the Table S2 in the Supporting Information.

**Figure 2:**
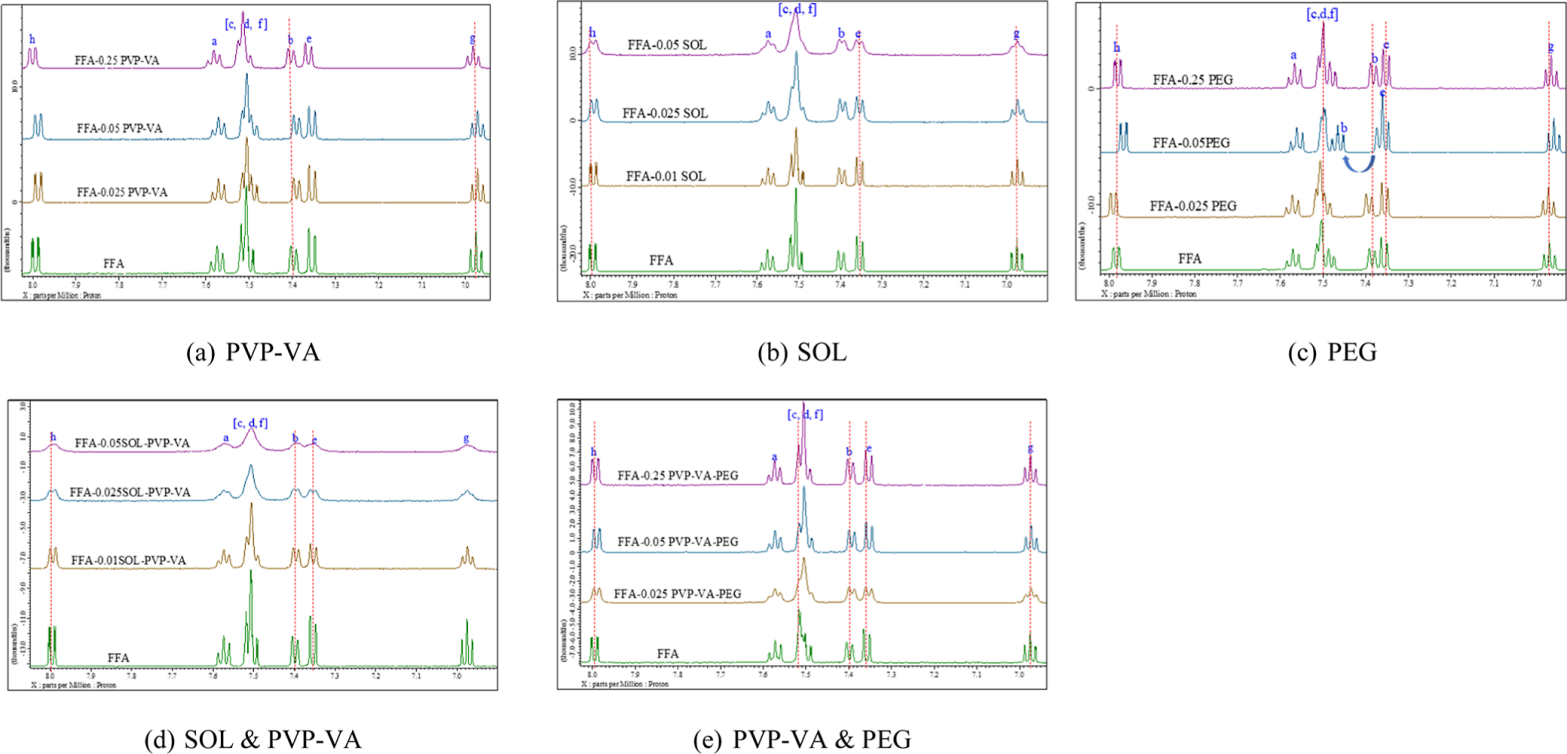
One-dimensional ^1^H NMR spectra analysis in FFA solution in the presence of a polymer or a combination: (a) PVP–VA; (b) SOL; (c) PEG; (d) SOL & PVP–VA; (e) PVP–VA & PEG.

PVP–VA caused upfield shifts of the H_b_ and H_g_ in Tables 2 and S2 in the Supporting Information. Although other peak positions remained unchanged, peak broadening with increasing polymer concentration was observed, particularly of H_h_ (7.35 ppm), the H_c_, multiplet (7.57–7.47 ppm), H_e_ (7.35 ppm) and H_g_ (6.98 ppm). These effects are shown in Figure 2a.

**Table 2:** Changes in the ^1^H Chemical Shifts of the Protons of FFA in Solution of FFA, FFA–TP, and FFA–NIC in the Presence and Absence of Polymers

polymer	concentration of individual polymer(s) (mg/mL)	FFA Δ chemical shift (ppm)					FFA–TP Δ chemical shift (ppm)					FFA–NIC Δ chemical shift (ppm)				
		ΔH_h_	ΔH_c_	ΔH_b_	ΔH_e_	ΔH_g_	ΔH_h_	ΔH_c_	ΔH_b_	ΔH_e_	ΔH_g_	ΔH_h_	ΔH_c_	ΔH_b_	ΔH_e_	ΔH_g_
PVP–VA	0.025	0.00	0.00	–0.01	0.00	–0.01	0.00	0.00	–0.01	0.00	0.00	0.00	0.00	–0.01	0.00	0.00
	0.05	0.00	0.00	–0.01	0.00	–0.01	–0.01	0.00	–0.01	0.00	0.00	0.00	0.00	–0.01	0.00	0.00
	0.25	0.00	0.00	0.00	0.00	–0.01	0.00	0.00	0.00	0.00	0.00	0.00	0.01	–0.01	0.00	0.00
SOL	0.01	–0.01	0.00	0.00	0.00	0.00	0.00	0.00	0.00	0.00	0.00	0.00	0.01	–0.01	0.00	0.00
	0.025	–0.01	0.00	0.00	0.00	–0.01	0.00	0.00	0.00	0.00	0.00	0.00	0.00	–0.01	0.00	–0.01
	0.05	–0.01	0.00	0.00	0.01	–0.01	0.00	0.00	0.01	0.01	0.00	0.00	0.01	0.00	0.02	0.00
PEG	0.025	0.01	0.01	0.01	0.00	0.00	0.00	0.00	–0.01	0.00	0.00	0.00	0.00	–0.02	–0.01	0.00
	0.05	–0.02	0.00	0.07	0.01	–0.01	0.00	0.00	0.00	0.00	0.00	0.00	0.00	–0.01	–0.01	0.00
	0.25	0.00	0.00	0.00	0.00	0.00	0.00	0.00	–0.01	0.00	0.00	0.00	0.00	0.00	–0.01	0.00
PVPVA–PEG	0.025	–0.01	0.00	–0.01	–0.01	–0.01	0.00	–0.01	–0.01	–0.01	0.00	0.00	0.00	–0.01	–0.01	–0.01
	0.05	–0.01	–0.01	–0.01	–0.01	–0.01	0.00	–0.01	–0.01	–0.01	0.00	0.00	–0.01	–0.01	–0.01	–0.01
	0.25	–0.01	0.00	0.00	–0.01	0.00	0.00	–0.01	–0.01	–0.01	0.00	0.00	0.00	–0.01	–0.01	–0.01
SOL-PVP–VA	0.01	–0.01	0.00	0.00	–0.01	0.00	0.00	–0.01	–0.01	0.00	0.00	0.00	–0.01	0.00	–0.01	0.00
	0.025	–0.01	0.00	0.00	–0.01	0.00	0.00	–0.01	–0.01	0.00	0.00	0.00	–0.01	0.00	–0.01	0.00
	0.05	–0.01	0.00	–0.02	0.01	0.00	0.00	–0.01	–0.01	0.02	0.00	0.00	0.00	0.00	0.01	0.00

SOL caused upfield shifts (Δ −0.01 ppm) in H_h_ (across all concentrations) and H_g_ (at 0.01 and 0.025 mg/mL). At higher concentrations (0.05 mg/mL), H_e_ exhibited a downfield shift (Δ +0.01 ppm). There was also significant (much more than PVP–VA) broadening of all peaks, which was enhanced with increasing polymer concentration resulting in merging peaks and altered coupling/splitting patterns, particularly at concentration above 0.025 mg/mL as shown in Figure 2b.

The presence of PEG influenced the chemical shifts of all the tracked protons. PEG induced a significant downfield shift in H_b_ (Δ +0.07 ppm) at 0.05 mg/mL, which then became part of the Hc multiplet, as shown in Figure 2c. H_e_ experienced a minor downfield shift (Δ +0.01 ppm) and an increase in splitting, transforming its doublet into a triplet. H_h_ and H_g_ experienced an upfield shift (Δ −0.02 and −0.01 ppm respectively) at 0.05 mg/mL. At 0.025 mg/mL, H_h_, H_b_ and H_c_ experienced a downfield shift (Δ +0.01 ppm).

The combination of PEG and PVP–VA primarily caused upfield shifts (Δ −0.01 ppm) in the FFA peaks (Tables 2 and S2 in the Supporting Information). Line broadening was observed only at the lowest (0.025 mg/mL) polymer concentration [Figure 2e].

In the presence of a combination of SOL and PVP–VA, a synergetic effect was observed, with line broadening starting at a low concentration (0.01 mg/mL) and increasing with concentration. At 0.05 mg/mL H_h_ and H_b_ shifted upfield (Δ −0.01 ppm and −0.02 ppm, respectively), while H_e_ shifted downfield (Δ +0.01 ppm).

The line broadening could indicate interactions due to physical adsorption, noncovalent interactions or changes in proton mobility (slower tumbling due to increased viscosity), all of which can cause positive cross-peaks in the NOESY spectra.

Additional details on the chemical shifts of the FFA and spectra are provided in the Supporting Information (Table S2, Figures S1 and S5).

#### 3.2.2 Effects of Polymers on the Chemical Shifts of FFA in the FFA–TP and FFA–NIC Solutions

The effects of polymers on FFA protons in cocrystal solutions are summarized in Table 2. Changes in chemical shifts (Δ ppm) ranged from 0.01 to 0.02, varying across protons and polymer concentrations. Significant observations included concentration-dependent line broadening in the presence of both single polymers and combinations. The effect was more pronounced with SOL and its combinations, which showed synergistic effects, starting at lower concentrations (0.01 mg/mL).

Unlike in FFA solutions, PEG in cocrystal solutions primarily affected chemical shifts without causing significant line broadening or changes in splitting patterns. Representative spectra for line broadening and chemical shift changes are provided in Figures S3 and S4 in the Supporting Information.

Overall, these findings indicate that polymers, both individually and in combinations, altered the chemical environment of FFA, potentially stabilizing supersaturation by modulating molecular interactions. Further details, can be found in Table S2, Figures S1 and S5 in the Supporting Information, which provide a comprehensive comparison of the chemical shifts across all conditions.

### 3.3 NOESY Spectra Analysis

**Figure 3:**
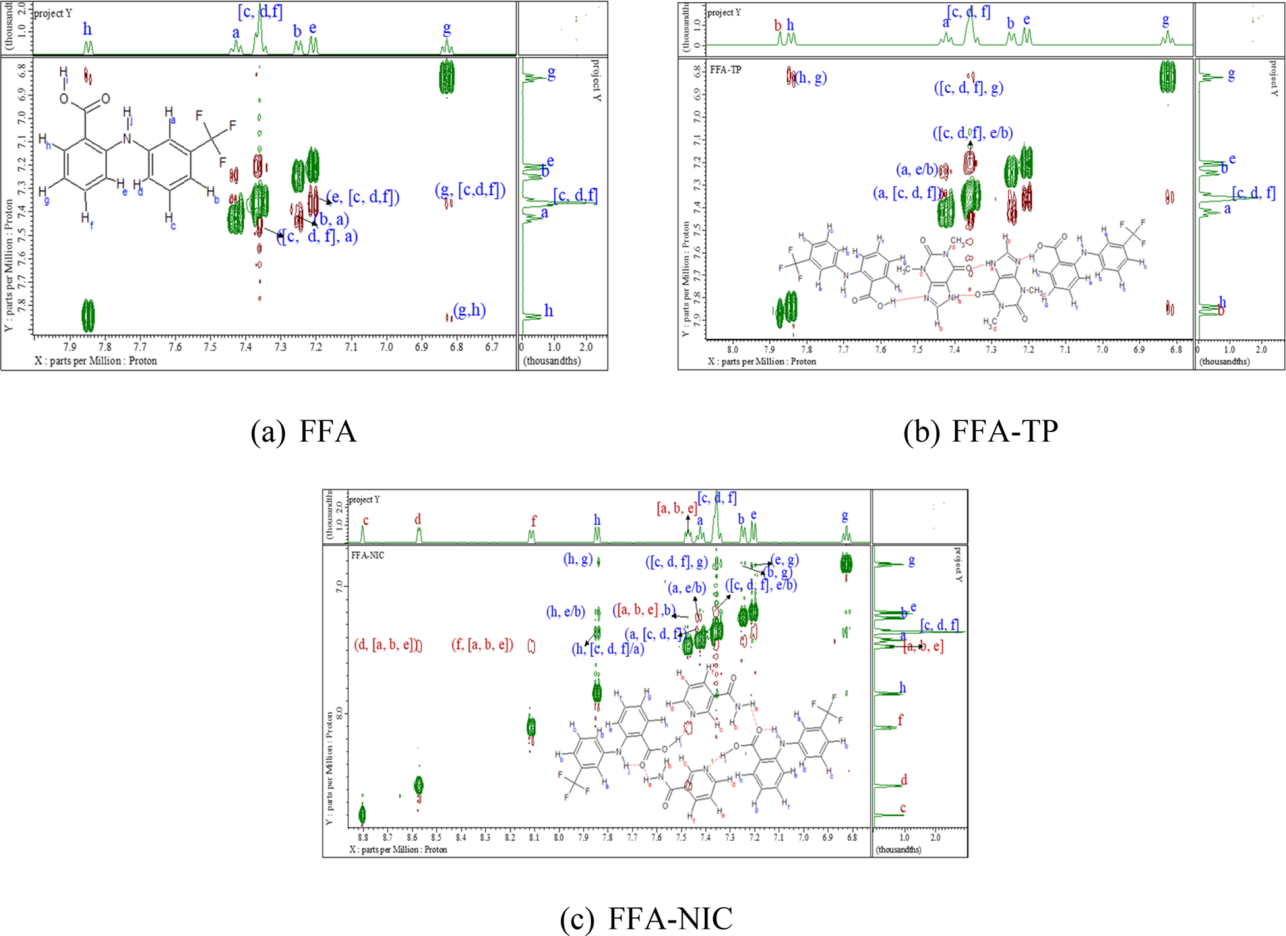
NOESY spectra of solutions of (a) FFA, (b) FFA–TP, and (c) FFA–NIC in the absence of polymers in DMS0_6_–D_2_O solvent.

The labels of the cross-peaks in the NOESY spectra in Figure 3 are color-coded. FFA 1D spectral peaks are labeled in blue while those of TP and NIC are labeled in red. A cross-peak between FFA and TP or NIC will have both blue and red letters corresponding to FFA and TP/NIC respectively. While self-correlation peaks between FFA protons will have only blue letters.

#### 3.3.1 Effects of Polymers on the Interaction of FFA Molecules in the FFA alone Solution

The NOESY spectra of FFA solution without polymer [Figure 3a] showed negative cross-peaks (opposite color from the diagonal) between the FFA protons, suggesting longer distances between FFA molecules and the absence of aggregations. All NOESY spectra in the presence of polymers are shown in Figure S6 in the Supporting Information.

**Figure 4:**
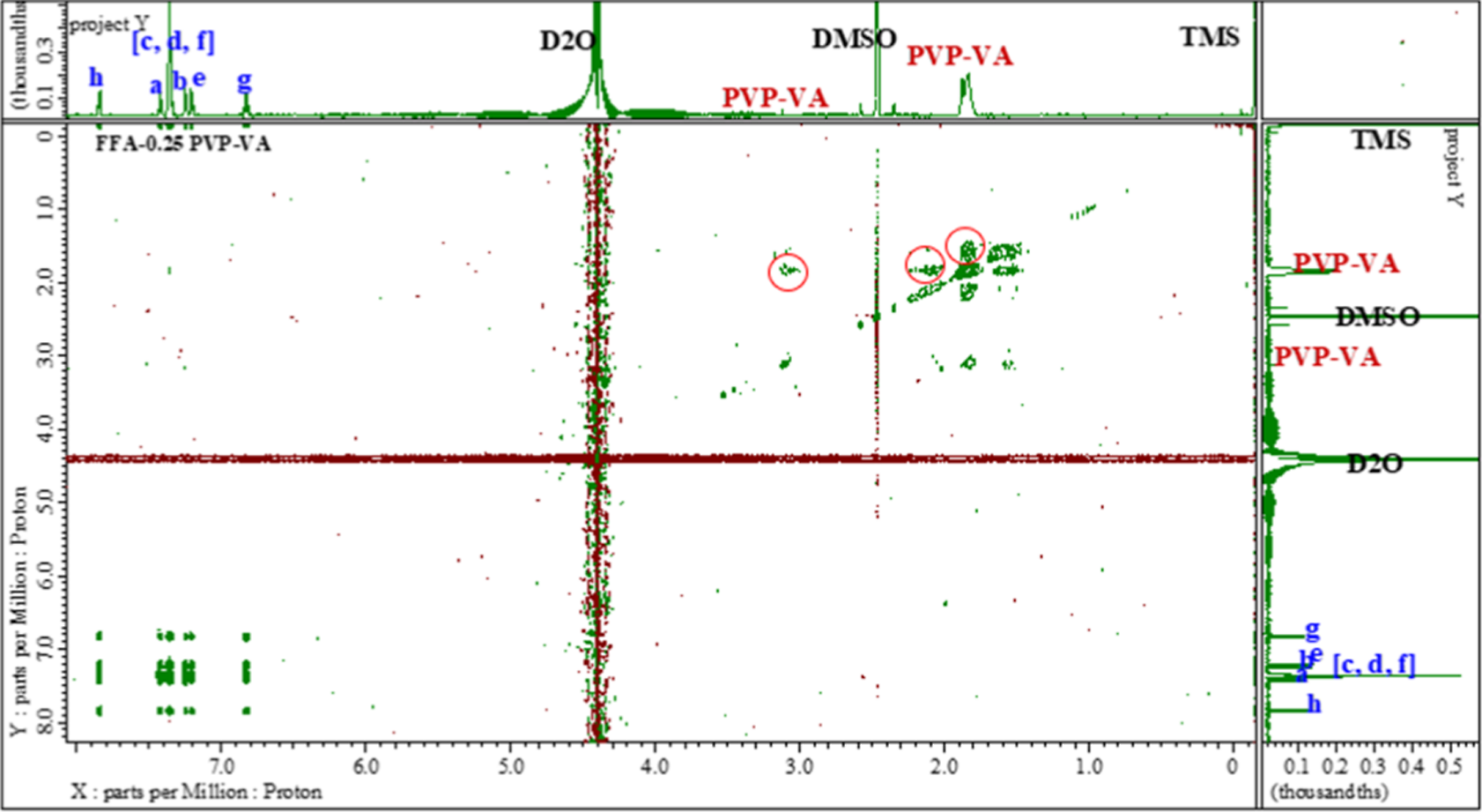
Full two-dimensional ^1^H/^1^H NMR spectra analysis of FFA solution in the presence of 0.25 mg/mL PVP–VA showing PVP–VA self-correlation peaks (in red circles).

With 0.025 mg/mL PVP–VA, cross-peaks between FFA protons became positive (same color as the diagonal) and increased in intensity, indicating reduced intermolecular distances. Additional cross-peaks appeared such as H_h_ and H_g_, H_e_ and H_c_ multiplets (Figure S6 in the Supporting Information). At 0.05 mg/mL PVP–VA, the number and intensity of the FFA–FFA cross-peaks further increased. However, at 0.25 mg/mL, the FFA–FFA cross-peaks intensity decreased and self-associated cross-peaks for PVP–VA were observed, indicating excessive polymer interactions (Figure 4). No FFA and PVP–VA cross-peaks were observed at any concentrations.

In the presence of SOL, the FFA–FFA cross-peak intensity increased with concentration but decreased at 0.05 mg/mL. All the cross-peaks remained positive and no FFA and SOL cross-peaks (Figure S6 in the Supporting Information).

At 0.025 mg/mL PEG, positive FFA–FFA cross-peaks were observed. At 0.05 and 0.25 mg/mL, some cross-peaks became negative with reduced intensity, suggesting PEG reduced the FFA self-association. For example, the cross-peak between H_b_ and H_g_ seen at lower concentrations was absent at 0.25 mg/mL. No FFA and PEG cross-peaks were detected.

In solutions containing both PEG and PVP–VA, all FFA–FFA cross peaks remained positive at all concentrations (Figure S4 in the Supporting Information). No FFA and polymer cross-peaks were observed. Cross-peaks intensity decreased at 0.05 mg/mL compared to 0.025 mg/mL, but increased again at 0.25 mg/mL exceeding the initial intensity.

In the presence of PVP–VA and SOL, only positive FFA–FFA cross-peaks were observed. Cross-peak intensity decreased with increasing polymer concentration.

#### 3.3.2 Effects of Polymers on the Interaction of FFA Molecules in the FFA–TP Solution

Overall, polymer addition modulated FFA–FFA interactions, primarily by altering the sign and intensity of cross-peaks. Correlations between TP and FFA or polymers were only observed in specific polymer-coformer systems. Representative spectra are shown in Figure S6 in the Supporting Information.

Without polymer, negative cross-peaks were observed between FFA protons while none appeared between FFA and TP [Figure 3b]. The addition of polymers altered the sign, number and intensity of FFA–FFA cross-peaks in a concentration-dependent manner. PVP–VA increased the number and intensity of positive FFA–FFA cross-peaks at all concentrations. At higher concentrations (0.25 mg/mL), correlations emerged between TP and PVP–VA, as well as between TP (H_b_) and FFA (H_c_). No cross-peaks were observed between FFA and PVP–VA.

SOL increased positive FFA–FFA cross-peaks with no correlations between FFA and SOL. Cross-peak intensity was slightly decreased with increasing SOL concentration.

PEG maintained negative FFA–FFA crosspeaks across all concentrations with reduced intensity compared to solution without polymer. No cross-peaks between polymer PEG with coformer (TP) or FFA were observed.

The combination of PVP–VA and SOL enhanced positive FFA–FFA cross peaks. However, the intensity of these contours decreased with increasing concentration. Correlations between TP (H_b_) and FFA protons were consistently observed. Increasing polymer concentration reduced cross-peak intensity.

With the combination of PVP–VA and PEG, positive FFA–FFA cross-peaks were observed, with intensity decreasing at higher polymer concentrations. At 0.25 mg/mL, self-correlation peaks appeared between PVP–VA and correlations between TP and FFA were detected.

#### 3.3.3 Effects of Polymers on the Interaction of FFA Molecules in the FFA–NIC Solution

FFA–NIC spectra revealed both negative and positive self-correlations for FFA, negative self-correlations for NIC, and a small cross-peak between FFA and NIC [Figure 3c], suggesting possible interactions between FFA and NIC.

At 0.025 mg/mL PVP–VA, all FFA–FFA correlations were positive and intense with a small positive FFA and NIC cross-peak and no NIC self-correlations. NIC self-correlation appeared at higher concentrations (0.05 and 0.25 mg/mL in Figure S6 in the Supporting Information).

SOL caused positive FFA–FFA correlations, negative NIC self-correlations, and a small positive cross-peak of FFA and NIC. At 0.05 mg/mL, cross-peaks appeared between NIC protons (a, b, c) and FFA (H_b_).

PEG showed negative self-correlations for FFA and NIC, and positive FFA–NIC correlations. At 0.025 mg/mL, FFA self-correlation peaks between H_e_ and H_g_ and between H_b_ and H_g_ were absent. At higher concentrations (0.05 and 0.25 mg/mL), H_e_ and H_g_ self-correlation for FFA appeared but the one between H_b_ and H_g_ remained absent. No FFA and PEG cross-peaks were observed.

The combination of PVP–VA and PEG showed high-intensity positive FFA–FFA correlations and negative NIC self-correlations without any FFA and NIC correlations.

The PVP-VA and SOL mixture caused high-intensity positive correlations for FFA and negative NIC self-correlations, with FFA–FFA cross-peak intensity decreasing at higher concentrations. At 0.05 mg/mL, a small cross-peak between FFA and NIC appeared, and at 0.25 mg/mL, positive PVP–VA self-correlations were detected. All spectra in the presence of polymers are represented in the Supporting Information (Figure S6).

### 3.4 DOSY Spectra Analysis

The DOSY NMR experiments were conducted to understand the diffusion properties of FFA in the absence/presence of different polymers in solution. The diffusion coefficient of FFA was determined based on the proton H_g_ (6.97 ppm) as it was prone to the change of the environment and intermolecular interaction with the other components in solution. DOSY spectra are presented in Figure S8 as two-dimensional (2D) plots with chemical shift (ppm) on the horizontal axis and D (10–10 m2/s) on the vertical axis in the Supporting Information.

A summary of the FFA apparent diffusion coefficients based on the characteristic proton H_g_ in solution in the presence of different polymers is shown in Table S3 in the Supporting Information. Due to the large amount of test samples, the experiments had to be conducted on different days over a longer period. To reduce the experimental errors, all of the samples in the presence of different concentrations of a polymer were freshly prepared using the newly prepared FFA or FFA cocrystal samples shown in Table S1 in the Supporting Information. It can be seen that the FFA diffusion coefficients of different FFA samples without a polymer were changing for the different preparations (Table S3 in the Supporting Information), although the FFA concentration was the same, which was most likely caused by the sample preparations. As the analysis was focused on the change of the FFA diffusion coefficients in the presence of a different concentration of a polymer relative to the solution without the polymer (called baseline data shown in Table S3 in the Supporting Information), the change of FFA diffusion coefficients can be avoided by the sample preparation shown above.

#### 3.4.1 Effects of Polymers on the Diffusion of FFA Molecules in the FFA alone Solution

PVP–VA consistently increased the diffusion coefficient of FFA across all concentrations, with the highest increase observed at 0.25 mg/mL, suggesting enhanced molecular mobility.

SOL showed a concentration-dependent effect, initially reducing the diffusion coefficient at lower concentrations but increasing it at 0.05 mg/mL, indicating potential interactions that facilitate diffusion at higher concentrations.

PEG generally had a smaller impact compared to PVP–VA and SOL, with slight increases in diffusion coefficients at some concentrations but reductions at others.

Polymer combinations such as PVP–VA–SOL and PVP–VA–PEG showed trends similar to their individual components for example PVP–VA–PEG trend was similar to PVP–VA and PVP–VA–SOL similar to SOL, with PVP–VA–SOL enhancing diffusion more significantly at higher concentrations.

#### 3.4.2 Effects of Polymers on the Interaction of FFA Molecules in the Coformer-Containing Solutions

In FFA–TP and FFA–NIC solutions, the PVP–VA and SOL consistently increased diffusion coefficients across all concentrations. PEG demonstrated a greater increase in FFA diffusion coefficients in the presence of coformers compared to FFA alone, with slight reductions at one concentration in both coformer systems. The PVP–VA–PEG combination generally reduced FFA diffusion coefficients, although an increase was observed at one concentration in each coformer system. The PVP–VA–SOL combination consistently increased FFA diffusion coefficients across all concentrations in FFA–NIC solutions but showed mixed effects in FFA–TP solutions, with an increase at one concentration and reductions at others.

Overall, the trends indicate that polymer effects on FFA diffusion are influenced by the presence of coformers, with combinations often showing synergistic or variable effects depending on the system. Detailed diffusion coefficient data and trends are described in the Supporting Information and provided in Table S3 and Figure S7.

### 3.5 Molecular Dynamics Simulations

The MD simulations examined interaction energies between two FFA molecules across various formulations focusing on the effects of polymers and their combinations in solutions containing neat FFA, FFA–TP, and FFA–NIC cocrystals. Interaction energies are analyzed in terms of hydrogen bonding, VDW, and electrostatic contributions, with total nonbond energies calculated from these to assess molecular interactions. VDW interactions emerged as the dominant contributor to total nonbond energy. These forces primarily induced repulsive effects, which counteracted the close packing of molecules and inhibited nucleation. Hydrogen bonding and electrostatic interactions, while smaller in magnitude, played significant roles in modulating the overall interaction energies.

In solutions containing neat FFA, polymers consistently increased the repulsive VDW energy across all of the formulations. This trend was most pronounced with combinations of SOL-PVP–VA and PEG–PVP–VA, where the increase in repulsive forces strongly correlated with the inhibition of nucleation, as seen in longer induction times and fewer nucleated samples shown in the nucleation experiments in Section 3.3. Among the single polymers, the inclusion of PEG led to the biggest increase in the VDW and the total nonbond energy.

Without polymer in the solution, the inclusion of a coformer of TP or NIC increased the VDW energy and the total nonbond energy indicating that the interaction between two FFA molecules became weaker. PEG can significantly increase VDW repulsion in the FFA–TP system, indicating the potential for strong inhibition of nucleation of FFA (Table 3). However, there was not much change in the VDW repulsions in the FFA–NIC system. PVP–VA increased VDW repulsion in both the FFA–TP and FFA–NIC systems. The extent of the increase was close in FFA and FFA–TP systems. SOL introduced a mixed effect on VDW interactions, reducing the repulsive forces in the FFA–NIC system but almost no effect on FFA–TP systems. This mixed effect could result in varying levels of nucleation inhibition of FFA in solution, i.e., with SOL showing stronger inhibitory effects in FFA–TP while being less effective in FFA–NIC where nucleation was relatively more favorable shown in the nucleation experiments in Section 3.3. PVP–VA–SOL exhibited the most substantial increase in repulsive VDW energy in the FFA–TP system, resulting in the strongest inhibition of nucleation. However, it resulted in a reduction of VDW repulsive forces in FFA–NIC which could indicate an increased number of nucleated samples and shorter induction times. PVP–VA–PEG also exhibited a substantial increase in repulsive VDW energy, particularly in the FFA system. However, it resulted in a moderate reduction of VDW repulsive forces in FFA–TP and FFA–NIC. The addition of PVP–VA with PEG seems to reduce the efficiency of PEG in inhibiting nucleation by reducing the repulsive forces and hence reducing the energy barrier compared to PEG alone.

**Table 3:** Interaction Energies (kcal/mol) between FFA Molecules in Each of the Tests

formulation	energy type	FFA		FFA–TP		FFA–NIC	
		contribution	energy Δ	contribution	energy Δ	contribution	energy Δ
no polymer	H-bonding	–0.093		0		–0.945	
	VDW	50.21		64.707		64.05	
	electrostatic	–4.858		–3.963		–3.535	
	total nonbond energy	45.252		60.738		59.563	
PEG	H-bonding	0	0.093	–0.001	–0.001	–0.007	0.938
	VDW	66.307	16.097	71.553	6.846	62.979	–1.071
	electrostatic	–5.253	–0.395	–5.154	–1.191	–2.628	0.907
	total nonbond energy	61.048	15.796	66.394	5.656	60.338	0.775
PVP–VA	H-bonding	0	0.093	–0.268	–0.268	0	0.945
	VDW	59.446	9.236	70.432	5.725	73.448	9.398
	electrostatic	–5.202	–0.344	–5.452	–1.489	–4.515	–0.98
	total nonbond energy	54.237	8.985	64.706	3.968	68.927	9.364
SOL	H-bonding	–0.191	–0.098	–1.181	–1.181	–0.028	0.917
	VDW	63.219	13.009	64.204	–0.503	51.167	–12.883
	electrostatic	–5.347	–0.489	–6.485	–2.522	–5.773	–2.238
	total nonbond energy	57.675	12.423	56.552	–4.186	45.36	–14.203
PVP–VA–SOL	H-bonding	–0.04	0.053	–0.735	–0.735	–0.473	0.472
	VDW	73.681	23.471	72.002	7.295	59.463	–4.587
	electrostatic	–5.985	–1.127	–6.442	–2.479	–5.76	–2.225
	total nonbond energy	67.65	22.398	64.819	4.081	53.224	–6.339
PVP–VA–PEG	H-bonding	–0.827	–0.734	0	0	–0.828	0.117
	van der Waals	68.59	18.38	62.155	–2.552	59.353	–4.697
	electrostatic	–5.501	–0.643	–5.523	–1.56	–6.563	–3.028
	total nonbond energy	62.256	17.004	56.625	–4.113	51.956	–7.607

The MD simulations also provided valuable insights into the evolution of the distance of the FFA molecules, FFA diffusion coefficients and RDF in the FFA, FFA–TP and FFA–NIC solutions in the absence and presence of a polymer or a combination. Further details can be found in Figures S9–S11 in the Supporting Information. The brief description is summarized below.

The speed of FFA aggregation was observed to be the fastest in the neat FFA solution, with the distance between FFA molecules decreasing to less than 5 Å (Å) by the end of the simulation, indicating significant aggregation. The addition of either a coformer (TP or NIC), a polymer (such as PVP–VA, PEG, or SoL), or a combination of both generally reduced the rate of FFA aggregation, as reflected by the reduced steepness of the distance evolution trendline. This is illustrated in the distance evolution graphs shown in Figure S9 in the Supporting Information, which suggests that the presence of coformers or polymers interferes with the close approach of FFA molecules, slowing down their aggregation. In the FFA solution with PVP–VA, however, the reduction in aggregation speed was minimal, with the distance between FFA molecules still showing a downward trend, though slightly moderated compared to the neat FFA solution. This indicates that PVP–VA alone was not particularly effective in reducing the aggregation speed of FFA molecules. In contrast, polymers like PEG and SOL, and especially combinations like PVP–VA–SOL, demonstrated a more pronounced reduction in aggregation speed, maintaining a larger average distance between FFA molecules throughout the simulation. Overall, the results suggest that the introduction of a coformer or certain polymers can act to moderate the aggregation behavior of FFA, likely by providing stabilizing interactions that prevent FFA molecules from coming into close proximity as rapidly as in the neat solution.

The RDF results (Figure S11 in the Supporting Information) indicated that neat FFA solutions exhibit strong aggregation tendencies, as demonstrated by a prominent peak below 5 Å with high *g*(*r*) values (approximately 20), which confirms close FFA–FFA contact and significant clustering. The introduction of coformers (TP or NIC) or polymers generally lowers the likelihood of FFA aggregation, as evidenced by a reduction in the FFA–FFA *g*(*r*) peak height and a shift to larger r distances. This shift suggests that FFA molecules are kept further apart, reducing the probability of close contact and aggregation. In the FFA solution, all polymers reduce the FFA–FFA peak height and/or increase the FFA–FFA distance, indicating a dispersion effect on FFA molecules, with PVP–VA–SOL being the most effective. In the FFA–TP solution, all polymers except PEG maintain FFA molecules at distances beyond 5 Å, indicating effective aggregation prevention in most cases. However, PEG exhibits a relatively smaller impact, allowing FFA molecules to remain at shorter distances. In the FFA–NIC solution, all polymers except SOL maintain FFA at larger distances, showing effective aggregation prevention. SOL appears to be less effective in this system, as it allows FFA molecules to remain at distances that could lead to aggregation. The RDF results provide a molecular-level picture of FFA aggregation tendencies that generally aligns with the experimental nucleation data, as both methods suggest that most polymers prevent FFA aggregation to a certain extent. However, discrepancies are noted with PEG in the FFA–TP solution. While the RDF results suggest that PEG does not reduce the FFA aggregation tendencies, the nucleation data (Table 4) indicate a stronger inhibitory effect.

**Table 4:** Numbers of the Nucleated Samples with the Averaged Induction Time

sample	FFA [nucleated samples/induction time (min)]	FFA–TP [nucleated samples/induction time (min)]	FFA–NIC [nucleated samples/induction time (min)]
no polymer	80/10.11	80/7.55	80/7.51
PVP–VA	80/7.36	78/24.52	80/10.73
PEG	8/83.18	13/104.00	28/99.63
SOL	80/18.08	33/95.10	77/34.85
PVP–VA–SOL	3/38.48	2/137.88	80/3.30
PVP–VA–PEG	50/79.85	36/95.24	66/49.87

The diffusion coefficient values, calculated from the MSD in the molecular dynamics simulations, showed that all polymers and combinations increased the diffusion coefficient of FFA in FFA–TP and FFA–NIC solutions compared to their respective no-polymer baselines (Figure S10 in the Supporting Information). The enhanced diffusion suggests greater mobility and dispersion of molecules, correlating with a reduced likelihood of aggregation in the simplified context of the MD simulations. In this context, diffusion enhancements are primarily interpreted as reduced interaction strength and dispersion, meaning that molecules are less likely to form stable, aggregation-prone contacts. While increased diffusion in simulations suggests reduced aggregation tendencies, this interpretation may differ from real-life experiments where enhanced diffusion can promote collisions and aggregation under certain conditions. This trend was observed in some of our DOSY results; for example, PVP–VA generally enhanced the diffusion coefficient of FFA in the DOSY results and was ineffective in sustaining the supersaturated state, as seen in the nucleation data (Table 4). In the MD simulations, only direct interactions between molecules were modeled, and complex solvation dynamics, such as micelle encapsulation (as seen with soluplus) or hydration shells (as observed with PEG), were not captured. This absence may lead to a simplified representation where increased diffusion implies a lower aggregation potential due to freer molecular movement. In the neat FFA solutions, however, the polymers exhibited varied effects on FFA diffusion. PEG and the combination of PVP–VA and SOL increased the diffusion coefficient, suggesting enhanced mobility and dispersion, while SOL, PVP–VA, and the combination of PVP–VA with PEG reduced the diffusion coefficient, indicating stabilization effects that may limit FFA movement. These findings highlight the complementary roles of MD simulations and experimental data in understanding the complex dynamics of FFA mobility and aggregation in solution, with simulation data providing insight into mobility trends and experimental results confirming the stabilizing effects that mitigate nucleation.

### 3.6 Nucleation Study

In each of the experiments, the number of nucleated samples and the induction time representing the averaged individual nucleated samples are shown in Table 4. In the Table, the nucleation data of FFA, FFA–TP and FFA–NIC solutions without polymer were taken from our previous publication.^41^

In the FFA solution, the addition of PVP–VA led to rapid nucleation, with all 80 samples nucleating and an induction time reduced to 7.36 min, indicating that PVP–VA accelerated the nucleation process. In contrast, SOL delayed the nucleation, with all 80 samples nucleating but an extended induction time of 18.08 min was achieved. PEG was a strong nucleation inhibitor, with only 8 out of 80 samples nucleating and an extended induction time of 83.18 min. The PVP–VA–PEG combination showed an effective inhibition effect, with only 3 samples nucleating and an induction time of 38.48 min, while the PVP–VA–SOL combination resulted in 50 nucleated samples with a longer induction time of 79.85 min.

In the FFA cocrystal solutions (FFA–TP and FFA–NIC), the addition of PVP–VA showed the highest nucleation activity, with all 80 samples nucleating in FFA–NIC and 78 samples in FFA–TP. However, the induction time for FFA–TP was much longer (24.52 min) compared to FFA–NIC (10.73 min). PEG acted as a nucleation inhibitor in both systems, with only 13 nucleated samples from FFA–TP (induction time 104.00 min) and 28 nucleated samples from FFA–NIC (induction time 99.63 min). Interestingly, SOL changed behavior in the cocrystal solutions, becoming an inhibitor with fewer nucleated samples than in the FFA solution: 33 samples for FFA–TP (induction time 95.10 min) and 77 samples for FFA–NIC (induction time 34.85 min). The PVP–VA–PEG combination was most effective in inhibiting nucleation in FFA–TP (only 2 samples nucleated, with an induction time of 137.88 min), but could not inhibit nucleation in FFA–NIC, where all 80 samples nucleated (induction time 3.30 min). The PVP–VA–SOL combination showed an inhibitory effect on FFA–TP and FFA–NIC, with 36 and 66 nucleated samples and induction times of 95.24 and 49.87 min, respectively.

## 4 Discussion

Pharmaceutical cocrystals offer a promising strategy to enhance drug compound solubility without altering the intrinsic pharmacological properties of the APIs, offering an alternative to traditional methods such as prodrugs. However, the supersaturated API concentration generated during cocrystal dissolution makes the API prone to nucleation and precipitation of the poorly soluble API crystals, potentially reducing the drug’s bioavailability and, in some cases, leading to therapeutic failure due to subtherapeutic dosing. Polymers PVP–VA, PEG and SOL studied in this work with many others such as polyvinylpyrrolidone (PVP) and hydroxypropyl methylcellulose (HPMC) have been investigated as potential inhibitors or moderators of nucleation, helping to sustain a supersaturated state and delay crystallization of the API in solution by modifying the interactions within the solution environment and stabilizing supersaturated solutions for a longer duration.

Despite these advances, the exact molecular interactions API, coformers, and polymers that govern nucleation dynamics in cocrystal systems remain unclear. The mechanisms by which polymers inhibit nucleation are complex and involve competing interactions during API precipitation. A deeper understanding of these interactions requires advanced experimental and computational approaches to analyze the nucleation process in detail.

This study explored the effect of polymeric excipients on the nucleation of FFA from its cocrystal solutions of FFA–TP and FFA–NIC. Using a combination of NMR (^1^H, DOSY and NOESY), MD simulations and nucleation studies using Crystal 16, we examined the molecular interactions influencing the FFA mobility and nucleation processes. These approaches provided critical insights into the nucleation process and optimizing cocrystal formulations for improved solubility, stability, and therapeutic efficacy. Here, we discussed the key findings and proposed the potential mechanisms for the observed behaviors due to inclusion of a polymer or a combination.

Polymers, even at higher concentrations, caused minimal perturbations in the ^1^H chemical shifts of FFA and its cocrystals, observed shifts within a narrow range (−0.02 to 0.07 ppm). This suggests weak to negligible interactions between the polymers and the drug or coformer protons, maintaining the structural integrity of the drugs and coformers in these conditions. However, significant line broadening resulting in changes in peak splitting patterns was observed particularly with SOL and its combination with PVP–VA in both FFA and its cocrystals, indicating reduced molecular mobility due to increased “friction” experienced by the drug molecules.^46^

Simulations further revealed that hydrogen bonding was not a major factor in the interactions between FFA and the polymers, contrary to common assumptions about polymer excipients. Instead, the Lennard-Jones (VDW) and Coulombic (electrostatic) interactions were the primary contributors to the supersaturated concentration of FFA in solution.

It is worth noting that the tested polymer concentrations were selected based on their solubility in the respective solvents used for the experiments. For the nucleation studies in Crystal 16, a concentration of 0.005 mg/mL was chosen as it represented the maximum soluble concentration of soluplus in the solvent, ensuring comparability across all polymers. Although other polymers had higher solubility, a uniform concentration was selected to allow direct comparison between single polymers and their combinations. Similarly, the polymer concentrations used in the NMR studies were determined based on their solubility in the NMR solvent. The concentration range was carefully selected to remain within the soluble limits, ensuring reliable and consistent measurements.

### 4.1 PVP–VA

In the nucleation studies, PVP–VA showed a marked acceleration of nucleation in the FFA–alone solutions, reducing the average induction time to 7.36 min. The addition of PVP–VA likely reduced the energy barrier for nucleation, resulting in faster crystallization. In the cocrystal solutions, PVP–VA only slightly extended the nucleation time by 16.97 and 3.22 min in FFA–TP and FFA–NIC solutions but eventually nucleation was observed in all or nearly all the vials. The NOESY spectra further revealed spatial proximity between FFA molecules, particularly for PVP–VA, which promoted the self-association of FFA molecules by facilitating molecular aggregation. The NOESY spectra also indicated API and coformer correlations which indicated that the polymer slightly maintained the FFA in interaction with coformer, however, the effect of enhanced mobility in FFA, as shown in the DOSY results, eventually promoted agglomeration due to collision.^47^

The MD simulations confirmed that in the presence of PVP–VA, FFA interacted through VDW forces and electrostatic interactions, rather than hydrogen bonding (Table 3). These nonbonded interactions keep FFA molecules dispersed, preventing premature aggregation and promoting diffusion. The RDF data (Figure S11 in the Supporting Information) also indicated that PVP–VA maintained a moderate distance between FFA molecules in FFA–TP and FFA–NIC solutions, balancing mobility with nucleation promotion. However, the DOSY NMR results showed that PVP–VA significantly increased the diffusion coefficients of FFA across all concentrations. This enhanced mobility surpassed the dispersive effect of VDW forces and resulted in the collision of FFA molecules which then triggered agglomeration. The distance between FFA molecules (Figure S9 in the Supporting Information Materials) was shown to reduce faster in the FFA–PVP–VA scenario than in the cocrystals-PVP–VA which aligns with the nucleation data (Table 4).

### 4.2 SOL

In the FFA solution, SOL delayed nucleation, increasing the induction time to 18.08 min (Table 4). Interestingly, in the FFA–TP and FFA–NIC formulations, SOL exhibited nucleation inhibition, further extending the induction times. These results suggest that SOL acted as a nucleation inhibitor, particularly in the cocrystal systems. Distance evolution in MD simulation (Figure S9 in the Supporting Information) revealed that FFA molecules spread apart in FFA–TP–SOL compared to FFA or FFA–NIC with SOL which was aligned with the nucleation data in Table 4. The MD simulations revealed that VDW forces and electrostatic interactions were the primary contributors to this stabilization of FFA structures.

While also increasing the diffusion coefficient, had a more moderate effect compared to PVP–VA. This increase indicated that SOL enhanced mobility but also stabilized FFA in solution, preventing rapid aggregation. The higher concentrations of SOL resulted in a more pronounced increase in diffusion, suggesting its role as both a mobility enhancer and stabilizer in solution (especially in FFA–TP where NOESY showed a decrease in intensity of FFA associated with increasing SOL concentration). The modest enhancement of diffusion with SOL could be attributed to the polymer’s amphiphilic nature, which allowed it to form micelle-like structures that encapsulate FFA molecules. Thus, the peak broadening observed in the ^1^H NMR results, despite increased diffusion (which is expected to cause sharp peaks), was likely due to the complex microenvironment created by SOL micelle. This was consistent with the behavior of drugs in polymeric micellar systems.^48^ The micelles restrict the drug’s internal motion even if the micelle as a whole might diffuse faster in solution leading to higher mobility on a macroscopic scale (as seen in the DOSY results).

### 4.3 PEG

PEG emerged as the most effective nucleation inhibitor among single polymers. In the FFA, FFA–TP and FFA–NIC systems, PEG extended the induction times by 73.07, 96.55, and 92.12 min, respectively.

In contrast to its strong nucleation inhibition, PEG had minimal effects on diffusion. The DOSY results showed that the diffusion coefficient of FFA in PEG solution increased only slightly to 0.5861 × 10^–10^ m^2^/s at 0.05 mg/mL, a marginal increase from the no-polymer baseline of 0.5835 × 10^–10^ m^2^/s. This suggested that PEG stabilized FFA molecules without significantly enhancing their mobility, supporting its role as a precipitation inhibitor through the reduction of molecular motion. This was likely due to PEG forming hydration shells around FFA molecules, which limited their mobility and reduced the probability of nucleation. These findings were consistent with previous studies showing PEG’s ability to stabilize supersaturated solutions by reducing molecular aggregation. PEG was also the only polymer that resulted in negative NOESY cross-peaks between the FFA peaks and also did not cause line broadening.

The MD simulations confirmed that PEG interacted with FFA mainly through steric stabilization, forming hydration shells that prevent FFA molecules from approaching each other closely enough to form nuclei. The RDF (Figure S11 in the Supporting Information) and distance evolution (Figure S9 in the Supporting Information) indicated that PEG maintained a large distance between FFA molecules, further inhibiting nucleation by reducing the likelihood of collisions.

### 4.4 PVP–VA–PEG

The combination of PVP–VA and PEG exhibited moderate nucleation inhibition compared to PEG alone. In the FFA–TP and FFA–NIC cocrystal systems, the PVP–VA–PEG combination resulted in longer induction times compared to no-polymer and PVP–VA alone, but it was not as effective as the PEG alone or PVP–VA–SOL combination. In the FFA–TP solution, the induction time was extended to 95.24 min with 36 nucleated samples, while in the FFA–NIC solution, the induction time was 49.87 min with 66 nucleated samples. The moderate inhibition suggested that the combination of PVP–VA and PEG tempered the nucleation rate, likely due to PEG’s ability to reduce molecular mobility combined with PVP–VA’s modulation of interactions. The NOESY spectra show that FFA cross-peaks become positive as opposed to being negative, as in the presence of PEG alone which shows that the FFA associates were closer in the presence of a combination of PVP–VA and PEG.

The DOSY results showed that the PVP–VA–PEG combination led to only moderate increases in diffusion and mainly reduced the diffusion coefficient. This indicated that PEG’s stabilizing effect limited the mobility-enhancing properties of PVP–VA, resulting in moderate diffusion enhancement but effective nucleation inhibition. The PVP–VA–PEG combination relied more heavily on PEG’s steric stabilization than on PVP–VA’s enhancement of molecular mobility. The distance evolution and RDF data indicated that PVP–VA–PEG kept the FFA molecules farther apart, preventing aggregation.

### 4.5 PVP–VA–SOL

The combination of PVP–VA and SOL exhibited synergistic effects in inhibiting nucleation, particularly in the case of FFA and FFA–TP cocrystals, where 3 samples of FFA solution nucleated after an induction time of 38 min and only 2 samples of FFA–TP solution nucleated after an induction time of 137.88 min. In contrast, the combination of PVP–VA and SOL accelerated the nucleation in FFA–NIC samples more than either polymer alone causing nucleation in all 80 vials after an induction time of 3.30 min.

The MD simulations revealed that in the presence of PVP–VA and SOL combination in the FFA and FFA–TP solutions FFA molecules formed stronger VDW interactions, enhancing stabilization. This was backed by the nucleation data in Table 4. In contrast, the VDW forces were reduced in FFA–NIC hence promoting aggregation. The DOSY results also showed the combination lowers diffusion of FFA molecules in the FFA and FFA–TP solution but increases diffusion in the FFA–NIC solution. This dual behavior underscored the role of coformers in determining the impact of polymer combinations on nucleation behavior.

The synergistic behavior of PVP–VA and SOL have been previously reported.^49^ Similar to the findings in this study, the researchers found that PVP–VA alone failed to encapsulate their study drug without SOL. PVP–VA was able to entrap the drug in the presence of SOL hence SOL was reported to aid PVP–VA in the encapsulation. The phenomenon was ascribed to possible intermolecular interaction between the polymers. However, the NOESY spectra in our study did not show any correlations between the polymers to support this claim.

## 5 Conclusion

This study demonstrated that the effects of polymers on the nucleation and diffusion behavior of flufenamic acid (FFA) and its cocrystals varied significantly, providing insights into how these polymers can be used to control crystallization and stability in pharmaceutical formulations. The combined use of NMR, Crystal 16 and MD simulations allowed us to investigate the molecular interactions and mechanisms behind these effects.

The results indicate that the mechanism of precipitation inhibition is closely linked to the molecular mobility and self-association behavior of FFA in the presence of polymers. While PVP–VA enhances molecular diffusion and accelerates nucleation, PEG acts as a potent nucleation inhibitor by stabilizing drug molecules and reducing their mobility. SOL strikes a balance by enhancing diffusion but delaying nucleation through micelle formation. The PVP–VA–PEG combination exhibits moderate nucleation inhibition, balancing mobility enhancement and stabilization, while the PVP–VA–SOL combination demonstrates more variable effects: accelerating nucleation in some systems (such as FFA–NIC) while delaying it in others (such as FFA–TP), depending on the interplay between mobility and stabilization. These findings underscore the potential of polymer combinations to finely tune the nucleation and supersaturation profiles of poorly soluble drugs, offering strategic options for optimizing crystallization and formulation stability.

From a thermodynamic perspective, PEG increases the free energy barrier to nucleation by reducing molecular mobility and preventing aggregation. This aligns with its role as a stabilizer, which has been observed in other pharmaceutical systems where PEG is used to delay crystallization. In contrast, PVP–VA reduces the free energy barrier by increasing the diffusion coefficients and promoting molecular interactions. The kinetic enhancement of nucleation observed in the presence of PVP–VA is likely due to the higher collision frequency of FFA molecules, leading to more frequent nucleation events.

## Supporting Information Available

NMR spectra (^1^H, DOSY and NOESY) of all components, molecular dynamics data visuals and additional experimental details including NMR sample preparation (PDF)
