# Supplementary material for: Insight into the Precipitation Inhibition of Polymers within Cocrystal Formulations in Solution Using Experimental and Molecular Modeling Techniques
Source: Cryst Growth Des. 2025 Feb 28;25(6):1799–812. doi: 10.1021/acs.cgd.4c01573 (PMC11926783; doi:10.1021/acs.cgd.4c01573)

Supporting Materials

Insight into the Precipitation Inhibition of Polymers within Cocrystal Formulations in Solution using Experimental and Molecular Modelling Techniques

Peace Alinda[†], *Adolfo Botana*[‡], *and Mingzhong Li**[,†]

[†]Leicester School of Pharmacy, De Montfort University, Leicester LE1 9BH, UK

[‡]JEOL (U.K.) LTD, Welwyn Garden City AL7 1LT, UK

Table S1: Details of NMR measurement samples along with preparation methods

**SINGLE POLYMER**		
**STOCK SOLUTIONS PREPARATION**		
**Desired sample Composition**	**A**	**B**
0.5mg/ml PEG or PVP-VA	3mg polymer	6ml of D20
0.1mg/ml PEG or PVP-VA	1ml of 0.5mg/ml polymer solution	4ml of D20
0.05mg/ml PEG or PVP-VA	2ml of 0.1mg/ml polymer solution	2ml of D20
0.1mg/ml SOL	0.6ml of 1mg/ml SOL	5.4ml of D20
0.05mg/ml SOL	2ml of 0.1mg/ml SOL	2ml of D20
0.02mg/ml SOL	0.8ml of 0.1mg/ml SOL	3.2ml of D20
1mg/ml FFA	4mg FFA	2ml of DMSO
1mg/ml FFA-0.64mg/ml TP	6.6mg FFA-TP cocrystals	4ml DMSO
1mg/ml FFA-0.2mg/ml NIC	5.7mg FFA-NIC cocrystals	4ml DMSO
**DILUTIONS**		
0.5mg/ml FFA	1ml of 1mg/ml FFA solution	1ml of D20
0.5mg/ml FFA-0.32mg/ml TP	1ml of 1FFA-0.64TP solution	1ml of D20
0.5 mg/ml FFA-0.1 mg/ml NIC	1ml of 1FFA-0.2NIC solution	1ml of D20
0.025 mg/ml PEG or PVPVA	1ml of 0.05ml/mg polymer solution	1ml DMSO
0.5 mg/ml FFA-0.25 mg/ml PEG or PVP-VA	1ml of 1mg/ml FFA solution	1ml of 0.5mg/ml polymer solution
0.5 mg/ml FFA -0.05 mg/ml PEG or PVP-VA	1ml of 1mg/ml FFA solution	1ml of 0.1mg/ml polymer solution
0.5 mg/ml FFA -0.025 mg/ml PEG or PVP-VA	1ml of 1mg/ml FFA solution	1ml of 0.05mg/ml polymer solution
0.5FFA-0.32TP-0.25PEG or PVP-VA	1ml of 1FFA-0.64TP solution	1ml of 0.5mg/ml polymer solution
0.5FFA-0.32TP-0.05PEG or PVP-VA	1ml of 1FFA-0.64TP solution	1ml of 0.1mg/ml polymer solution
0.5FFA-0.32TP-0.025PEG or PVP-VA	1ml of 1FFA-0.64TP solution	1ml of 0.05mg/ml polymer solution
0.5FFA-0.1NIC-0.25PEG or PVP-VA	1ml of 1FFA-0.2NIC solution	1ml of 0.5mg/ml polymer solution
0.5FFA-0.1 NIC -0.05PEG or PVP-VA	1ml of 1FFA-0.2NIC solution	1ml of 0.1mg/ml polymer solution
0.5FFA-0.1 NIC -0.025PEG or PVP-VA	1ml of 1FFA-0.2NIC solution	1ml of 0.05mg/ml polymer solution
0.025 mg/ml SOL	1ml of 0.05ml SOL solution	1ml DMSO
0.5 mg/ml FFA-0.05 mg/ml SOL	1ml of 1mg/ml FFA solution	1ml of 0.1mg/ml SOL
0.5 mg/ml FFA-0.025 mg/ml SOL	1ml of 1mg/ml FFA solution	1ml of 0.05mg/ml SOL
0.5 mg/ml FFA-0.01 mg/ml SOL	1ml of 1mg/ml FFA solution	1ml of 0.02mg/ml SOL
0.5 mg/ml FFA-0.32 mg/ml TP-0.05 SOL	1ml of 1FFA-0.64TP solution	1ml of 0.1mg/ml SOL
0.5FFA-0.32TP-0.025 SOL	1ml of 1FFA-0.64TP solution	1ml of 0.05mg/ml SOL
0.5FFA-0.32TP-0.01 SOL	1ml of 1FFA-0.64TP solution	1ml of 0.02mg/ml SOL
0.5FFA-0.1NIC-0.05 SOL	1ml of 1FFA-0.1NIC solution	1ml of 0.1mg/ml SOL
0.5FFA-0.1NIC-0.025 SOL	1ml of 1FFA-0.1NIC solution	1ml of 0.05mg/ml SOL
0.5FFA-0.1NIC-0.01 SOL	1ml of 1FFA-0.1NIC solution	1ml of 0.02mg/ml SOL
**POLYMER MIXTURE**		
**STOCK SOLUTIONS PREPARATION**		
**Desired sample Composition**	**A**	**B**
0.1mg/ml SOL-0.1mg/ml PVPVA	0.6ml of 1mg/ml SOL-PVPVA solution	5.4ml of D20
0.05mg/ml SOL-0.05mg/ml PVPVA	2ml of 0.1mg/ml SOL-PVPVA solution	2ml of D20
0.025mg/ml SOL-0.025mg/ml PVPVA	0.8ml of 0.1mg/ml SOL-PVPVA solution	3.2ml of D20
0.5mg/ml PEG-0.5mg/ml PVP-VA	3mg PEG & 3mg PVP-VA	6ml of D20
0.1mg/ml PEG-0.1mg/ml PVP-VA	1ml of 0.5mg/ml PEG-PVPVA solution	4ml of D20
0.05mg/ml PEG or PVP-VA	2ml of 0.1mg/ml PEG-PVPVA solution	2ml of D20
**DILUTIONS**		
0.5 mg/ml FFA-0.05 mg/ml SOL-PVPVA	1ml of 1mg/ml FFA solution	1ml of 0.1mg/ml SOL-PVPVA solution
0.5 mg/ml FFA-0.025 mg/ml SOL-PVPVA	1ml of 1mg/ml FFA solution	1ml of 0.05mg/ml SOL-PVPVA solution
0.5 mg/ml FFA-0.01 mg/ml SOL-PVPVA	1ml of 1mg/ml FFA	1ml of 0.02mg/ml SOL-PVPVA solution
0.5FFA-0.32TP-0.05 SOL-PVPVA	1ml of 1FFA-0.64TP	1ml of 0.1mg/ml SOL-PVPVA solution
0.5FFA-0.32TP-0.025 SOL-PVPVA	1ml of 1FFA-0.64TP solution	1ml of 0.05mg/ml SOL-PVPVA solution
0.5FFA-0.32TP-0.01 SOL-PVPVA	1ml of 1FFA-0.64TP solution	1ml of 0.02mg/ml SOL-PVPVA solution
0.5FFA-0.1NIC-0.05 SOL-PVPVA	1ml of 1FFA-0.1NIC solution	1ml of 0.1mg/ml SOL-PVPVA solution
0.5FFA-0.1NIC-0.025 SOL-PVPVA	1ml of 1FFA-0.1NIC solution	1ml of 0.05mg/ml SOL-PVPVA solution
0.5FFA-0.1NIC-0.01 SOL-PVPVA	1ml of 1FFA-0.1NIC solution	1ml of 0.02mg/ml SOL-PVPVA solution
0.025 mg/ml SOL-.025 mg/ml PVPVA	1ml of 0.05ml SOL-PVPVA solution	1ml DMSO
0.5FFA-0.05 SOL-PVPVA	1ml of 1mg/ml FFA solution	1ml of 0.1mg/ml SOL-PVPVA solution
0.025 PVPVA- PEG	1ml of 0.05ml/mg PVPVA- PEG solution	1ml DMSO
0.5FFA-0.25 PVPVA- PEG	1ml of 1mg/ml FFA solution	1ml of 0.5mg/ml PVPVA- PEG solution
0.5FFA-0.05 PVPVA- PEG	1ml of 1mg/ml FFA solution	1ml of 0.1mg/ml PVPVA- PEG solution
0.5FFA-0.025 PVPVA- PEG	1ml of 1mg/ml FFA solution	1ml of 0.05mg/ml PVPVA- PEG solution
0.5FFA-0.32TP-0.25 PVPVA- PEG	1ml of 1FFA-0.64TP solution	1ml of 0.5mg/ml PVPVA- PEG solution
0.5FFA-0.32TP-0.05 PVPVA- PEG	1ml of 1FFA-0.64TP solution	1ml of 0.1mg/ml PVPVA- PEG solution
0.5FFA-0.32TP-0.025 PVPVA- PEG	1ml of 1FFA-0.64TP solution	1ml of 0.05mg/ml PVPVA- PEG solution
0.5FFA-0.1NIC-0.25 PVPVA- PEG	1ml of 1FFA-0.2NIC solution	1ml of 0.5mg/ml PVPVA- PEG solution
0.5FFA-0.1 NIC -0.05 PVPVA- PEG	1ml of 1FFA-0.2NIC solution	1ml of 0.1mg/ml PVPVA- PEG solution
0.5FFA-0.1 NIC -0.025 PVPVA- PEG	1ml of 1FFA-0.2NIC solution	1ml of 0.05mg/ml PVPVA- PEG solution

Table S2: Comparison of the ^{1}H chemical shifts of the protons of FFA in solution of FFA, FFA-TP, and FFA-NIC in the presence and absence of polymers.

Polymer	Concentration of individual polymer(s) (mg/mL)	FFA Chemical shift (ppm)					FFA-TP Chemical shift (ppm)					FFA-NIC Chemical shift (ppm)				
		H_h	H_c	H_b	H_e	H_g	H_h	H_c	H_b	H_e	H_g	H_h	H_c	H_b	H_e	H_g
**PVP-VA**	0	7.99	7.51	7.40	7.36	6.98	7.99	7.51	7.40	7.35	6.97	7.99	7.50	7.40	7.35	6.97
	0.025	7.99	7.51	7.39	7.36	6.97	7.99	7.51	7.39	7.35	6.97	7.99	7.50	7.39	7.35	6.97
	0.05	7.99	7.51	7.39	7.36	6.97	7.98	7.51	7.39	7.35	6.97	7.99	7.50	7.39	7.35	6.97
	0.25	7.99	7.51	7.40	7.36	6.97	7.99	7.51	7.40	7.35	6.97	7.99	7.51	7.39	7.35	6.97
**SOL**	0	8.00	7.51	7.40	7.35	6.98	7.99	7.51	7.39	7.35	6.97	7.99	7.50	7.40	7.35	6.98
	0.01	7.99	7.51	7.40	7.35	6.98	7.99	7.51	7.39	7.35	6.97	7.99	7.51	7.39	7.35	6.98
	0.025	7.99	7.51	7.40	7.35	6.97	7.99	7.51	7.39	7.35	6.97	7.99	7.50	7.39	7.35	6.97
	0.05	7.99	7.51	7.40	7.36	6.97	7.99	7.51	7.40	7.36	6.97	7.99	7.51	7.40	7.37	6.98
**PEG**	0	7.98	7.50	7.38	7.35	6.97	7.98	7.50	7.39	7.35	6.97	7.99	7.51	7.40	7.36	6.97
	0.025	7.99	7.51	7.39	7.35	6.97	7.98	7.50	7.38	7.35	6.97	7.99	7.51	7.38	7.35	6.97
	0.05	7.96	7.50	7.45	7.36	6.96	7.98	7.50	7.39	7.35	6.97	7.99	7.51	7.39	7.35	6.97
	0.25	7.98	7.50	7.38	7.35	6.97	7.98	7.50	7.38	7.35	6.97	7.99	7.51	7.40	7.35	6.97
**PVP-VA-PEG**	0	8.00	7.51	7.40	7.36	6.98	7.99	7.51	7.40	7.36	6.97	7.99	7.51	7.40	7.36	6.98
	0.025	7.99	7.51	7.39	7.35	6.97	7.99	7.50	7.39	7.35	6.97	7.99	7.51	7.39	7.35	6.97
	0.05	7.99	7.50	7.39	7.35	6.97	7.99	7.50	7.39	7.35	6.97	7.99	7.50	7.39	7.35	6.97
	0.25	7.99	7.51	7.40	7.35	6.98	7.99	7.50	7.39	7.35	6.97	7.99	7.51	7.39	7.35	6.97
**SOL-PVP-VA**	0	8.00	7.51	7.40	7.36	6.97	7.99	7.51	7.40	7.35	6.97	7.99	7.51	7.39	7.36	6.97
	0.01	7.99	7.51	7.40	7.35	6.97	7.99	7.50	7.39	7.35	6.97	7.99	7.50	7.39	7.35	6.97
	0.025	7.99	7.51	7.40	7.35	6.97	7.99	7.50	7.39	7.35	6.97	7.99	7.50	7.39	7.35	6.97
	0.05	7.99	7.51	7.38	7.37	6.97	7.99	7.50	7.39	7.37	6.97	7.99	7.51	7.39	7.37	6.97

Table S3: Changes in the diffusion coefficient of proton H_g of FFA in FFA, FFA-TP and FFA-NIC solutions in the presence and absence of polymers

Sample	**FFA**					**FFA-TP**					**FFA-NIC**				
	Diffusion coefficient (x10-10m2/s)	measurement error	TMS Diffusion coefficient (x10-10m2/s)	FFA diff/TMS diff	change in FFA diff/TMS diff ratio relative to the solution without polymer	Diffusion coefficient (x10-10m2/s)	measurement error	TMS Diffusion coefficient (x10-10m2/s)	FFA diff/TMS diff	change in FFA diff/TMS diff ratio relative to the solution without polymer	Diffusion coefficient (x10-10m2/s)	measurement error	TMS Diffusion coefficient (x10-10m2/s)	FFA diff/TMS diff	change in FFA diff/TMS diff ratio relative to the solution without polymer
Baseline: no polymer	1.22087	0.07025	2.79051	0.43751	-	1.47677	0.07673	3.00092	0.49211	-	1.30491	0.14679	3.08919	0.42241	-
0.025 PVP-VA	1.59568	0.04484	2.82532	0.56478	0.12727	1.53206	0.10517	2.82363	0.54259	0.05048	1.58173	0.094	3.04007	0.52029	0.09788
0.05 PVP-VA	1.65412	0.15992	2.91108	0.56822	0.13071	1.53307	0.10505	2.82221	0.54322	0.05111	1.71859	0.07295	2.85928	0.60106	0.17865
0.25 PVP-VA	1.83734	0.43743	2.81845	0.65190	0.21439	1.62601	0.1455	2.81857	0.57689	0.08479	1.48151	0.17104	3.34494	0.44291	0.02050
Baseline: no polymer	1.44474	0.04387	2.63154	0.54901	-	1.19501	0.194	2.88181	0.41467	-	1.4176	0.04546	2.70227	0.52460	-
0.01 SOL	1.50936	0.10264	2.90597	0.51940	-0.02961	1.67978	0.10596	2.80889	0.59802	0.18335	1.65893	0.08894	2.75918	0.60124	0.07664
0.025 SOL	1.50251	0.16259	2.77078	0.54227	-0.00674	1.13428	0.22083	2.68983	0.42169	0.00702	1.47428	0.12567	2.65818	0.55462	0.03002
0.05 SOL	1.88875	0.42035	2.57076	0.73470	0.18570	1.73261	1.66152	2.77345	0.62471	0.21004	1.89899	0.30667	2.62604	0.72314	0.19854
Baseline: no polymer	1.73172	0.25904	2.96780	0.58350	-	1.45311	0.13532	2.83209	0.51309	-	1.57625	0.07115	2.98691	0.52772	-
0.025 PEG	1.51982	0.13672	2.76389	0.54988	-0.03362	1.39173	0.07155	2.43927	0.57055	0.05746	1.50507	0.09541	2.78333	0.54074	0.01303

0.05 PEG	1.62215	0.13628	2.76788	0.58606	0.00256	1.72287	0.14653	2.95365	0.58330	0.07021	1.46905	0.05371	2.80134	0.52441	-0.00331
0.25 PEG	1.61988	0.0777	2.86798	0.56482	-0.01869	1.33174	0.21686	3.11624	0.42735	-0.08573	1.53842	0.08498	2.78141	0.55311	0.02539
Baseline: no polymer	1.56325	0.02001	3.05370	0.51192	-	1.61406	0.01558	3.00690	0.53679	-	1.60114	0.07326	2.99084	0.53535	-
0.025 PVPVA-PEG	1.62995	0.04191	2.97811	0.54731	0.03539	1.58189	0.00828	2.86234	0.55266	0.01587	1.66302	0.01298	3.14805	0.52827	-0.00708
0.05 PVPVA-PEG	1.50526	0.01215	2.80196	0.53722	0.02530	1.39084	0.06377	2.99052	0.46508	-0.07170	1.67764	0.01296	2.95679	0.56739	0.03204
0.25 PVPVA-PEG	1.63852	0.01473	2.94683	0.55603	0.04411	1.58487	0.02981	3.03476	0.52224	-0.01455	1.43432	0.02064	2.77855	0.51621	-0.01914
Baseline: no polymer	1.67023	0.00928	3.01944	0.55316	-	1.79759	0.01317	3.10120	0.57964	-	1.64223	0.01543	2.97619	0.55179	-
0.01 SOL-PVPVA	1.59499	0.0985	2.98904	0.53361	-0.01955	1.65799	0.03809	2.89637	0.57244	-0.00721	1.57562	0.02704	2.80457	0.56180	0.01002
0.025 SOL-PVPVA	1.59821	0.0456	2.82467	0.56580	0.01265	1.83466	0.14483	2.98460	0.61471	0.03507	1.78483	0.11353	2.77538	0.64309	0.09130
0.05 SOL-PVPVA	1.94665	0.39409	2.59579	0.74993	0.19677	1.63853	0.11904	2.88724	0.56751	-0.01214	1.72448	0.0918	2.64208	0.65270	0.10091

Description of effects of polymers on the diffusion coefficient of FFA molecules

(1) Effects of polymers on the diffusion of FFA molecules in the FFA alone solution

Upon adding PVP-VA, the diffusion coefficient of FFA increased across all concentrations, with the highest diffusion coefficient observed at 0.25 PVP-VA (0.6519×10^{-10} m²/s). This represented a significant increase from the no-polymer baseline (0.4375×10^{-10} m²/s) for the PVP-VA experiments, indicating enhanced molecular mobility.

For SOL, the highest diffusion coefficient of FFA was observed at 0.05 mg/mL SOL (0.7347 × 10^{-10} m^2/s), representing an increase from the no-polymer baseline (0.5490 × 10^{-10} m^2/s) for the SOL experiments. However, this increase was more moderate compared to the effect of PVP-VA. Although the lower concentrations lowered the diffusion coefficient relative to the baseline, the diffusion coefficient of FFA increased with an increase in SOL concentration until it exceeded the baseline diffusion coefficient at 0.05 mg/mL SOL.

For PEG, the diffusion coefficient of FFA increased to 0.5861 × 10^{-10} m^2/s at 0.05 mg/mL PEG, which is similar to the no-polymer baseline for the PEG experiments (0.5835 × 10^{-10} m^2/s), indicating that PEG had a smaller effect on enhancing diffusion for FFA if it did enhance it. However, PEG generally reduced the diffusion coefficient as seen at 0.025 mg/mL and 0.25 mg/mL PEG.

The diffusion coefficient of FFA increased with an increase in the concentration of PVP-VA-PEG. At the highest concentration of 0.25 mg/mL PVP-VA-PEG, the diffusion coefficient increased to 0.5560 × 10^{-10} m^2/s, indicating a moderate increase from the no-polymer baseline (0.5119 × 10^{-10} m^2/s). This suggested that the combination enhances diffusion, though not as significantly as PVP-VA alone.

In the presence of PVP-VA-SOL, the trend was similar to SOL alone where the diffusion coefficient at the lowest concentration, 0.01 mg/mL PVP-VA-SOL (0.53361 × 10^{-10} m^2/s) was lower than the no-polymer baseline (0.5532 × 10^{-10} m^2/s) but then increases with increase in polymer concentration until it exceeded the baseline at the 0.05 mg/mL PVP-VA-SOL (0.7499 × 10^{-10} m^2/s). This large increase indicated that the combination of PVP-VA and SOL significantly enhanced FFA mobility at higher concentrations.

(2) Effects of polymers on the interaction of FFA molecules in the FFA-TP solution

In the presence of PVP-VA and just like the FFA solution, the diffusion coefficient of FFA increased across all the concentrations. The highest diffusion coefficient was observed at 0.25 mg/mL PVP-VA (0.5769 × 10^{-10} m^2/s), an increase from the no-polymer baseline (0.4921 × 10^{-10} m^2/s), indicating that PVP-VA enhances the diffusion of FFA-TP.

In the presence of SOL, the FFA diffusion coefficient increased across all concentrations and to 0.6247 × 10^{-10} m^2/s at 0.05 SOL, which was significantly higher than the no-polymer baseline for SOL experiments (0.4147 × 10^{-10} m^2/s), indicating a stronger effect on mobility for FFA-TP compared to FFA.

In the presence of PEG, the FFA diffusion coefficient increased slightly to 0.5833 × 10^{-10} m^2/s at 0.05 mg/mL PEG, compared to the no-polymer baseline for PEG experiments (0.5131 × 10^{-10} m^2/s). The diffusion coefficient was reduced to 0.42735 × 10^{-10} m^2/s at the highest concentration 0.25 mg/mL PEG.

In the presence of PVP-VA-PEG, the diffusion coefficient of FFA in FFA-TP increased slightly to 0.55266 × 10^{-10} m^2/s at 0.025 mg/mL PVP-VA-PEG, showing a small increase compared to the no-polymer baseline of 0.5368 × 10^{-10} m^2/s. This suggested that the combination of PVP-VA-PEG did not significantly enhance diffusion for FFA-TP. The diffusion coefficient in fact reduced below baseline to 0.46508 × 10^{-10} m^2/s at 0.05 mg/mL PVP-VA-PEG and to 0.52224 × 10^{-10} m^2/s at 0.25 mg/mL PVP-VA-PEG.

In the presence of PVP-VA-SOL, the highest diffusion coefficient was observed at 0.025 mg/mL PVP-VA-SOL (0.61471 × 10^{-10} m^2/s), which was a moderate increase from the no-polymer baseline of 0.57964 × 10^{-10} m^2/s. There was a minor reduction to 0.57244 × 10^{-10} m^2/s at 0.01 mg/mL PVP-VA-SOL which was similar to the no polymer baseline and an even greater reduction to 0.56751 × 10^{-10} m^2/s at 0.05 mg/mL PVPVA-SOL.

(3) Effects of polymers on the interaction of FFA molecules in the FFA-NIC solution

Similar to FFA and FFA-TP, the presence of PVP-VA increased the diffusion coefficient across all the concentrations. The diffusion coefficient increased to 0.6011×10^{-10} m²/s at 0.05 mg/mL PVP-VA, representing the most notable increase from the no-polymer baseline for the PVP-VA experiments (0.4224×10^{-10} m²/s), indicating enhanced mobility.

Similar to FFA-TP, the presence of SOL increased the diffusion coefficient across all the concentrations. The diffusion coefficient increased to 0.7231×10^{-10} m²/s at 0.05 SOL, compared to the no-polymer baseline for SOL experiments (0.5246×10^{-10} m²/s), indicating a stronger effect on mobility.

In the presence of PEG, the diffusion coefficient increased slightly to 0.5531×10^{-10} m²/s at 0.25 mg/mL PEG, compared to the no-polymer baseline for PEG experiments (0.5277×10^{-10} m²/s). There was a slight reduction to 0.52441×10^{-10} m²/s at 0.05 mg/mL PEG. But generally, PEG slightly increased the diffusion coefficient.

In the presence of PVPVA-PEG, the diffusion coefficient increased to 0.5674×10^{-10} m²/s at 0.05 mg/mL PVP-VA-PEG, representing an increase from the baseline of 0.5353×10^{-10} m²/s. But generally, the diffusion coefficient reduced to 0.52827×10^{-10} m²/s at 0.025 mg/mL PVP-VA-PEG and to 0.51621×10^{-10} m²/s at 0.25 mg/mL PVP-VA-PEG. This suggested that the combination reduced the mobility of FFA-NIC in solution.

In the presence of PVP-VA-SOL, the highest diffusion coefficient was observed at 0.25 mg/mL PVP-VA-SOL (0.6527×10^{-10} m²/s), which was a significant increase from the no-polymer baseline of 0.55179×10^{-10} m²/s. Like in the presence of PVP-VA or SOL alone, the diffusion coefficient increased across all the concentrations and it also increased with increasing concentration. This indicated that PVP-VA-SOL significantly enhances diffusion for FFA-NIC

Figure S1: Showing the peak positions (ppm) of FFA protons (H_h, H_c, H_b, H_e, H_g) in solutions of (a) FFA, (b) FFA-TP, and (c) FFA-NIC in the absence and presence of varying polymers concentrations.

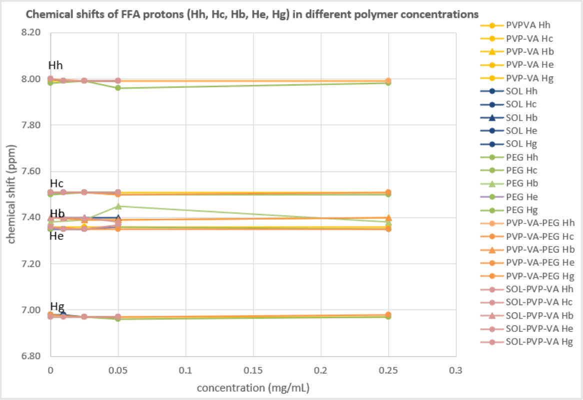

(a) FFA

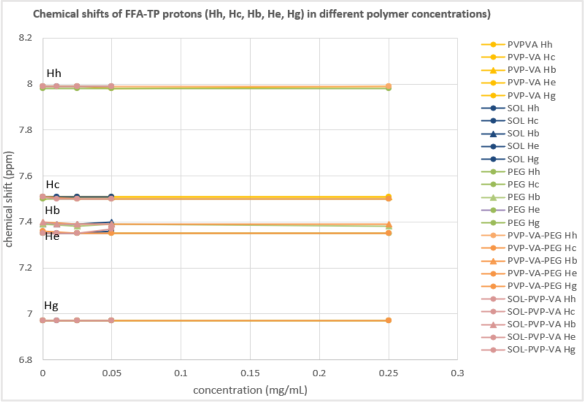

(b) FFA-TP

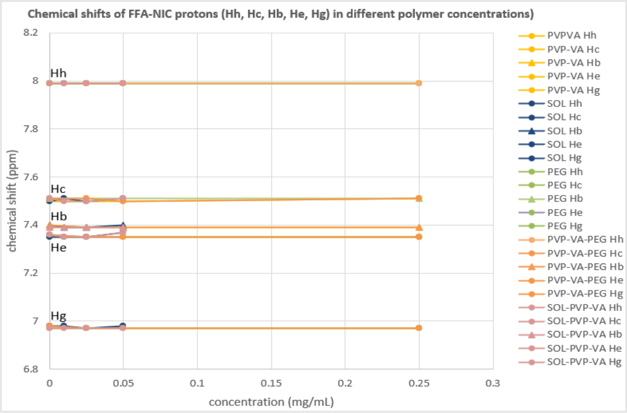

(c) FFA-NIC

Figure S2: Assignments of the ^{1}H chemical shifts of the protons of FFA (a), FFA-TP (b), FFA-NIC (c), PEG (d), PVP-VA (e), SOL (f)

(a) FFA

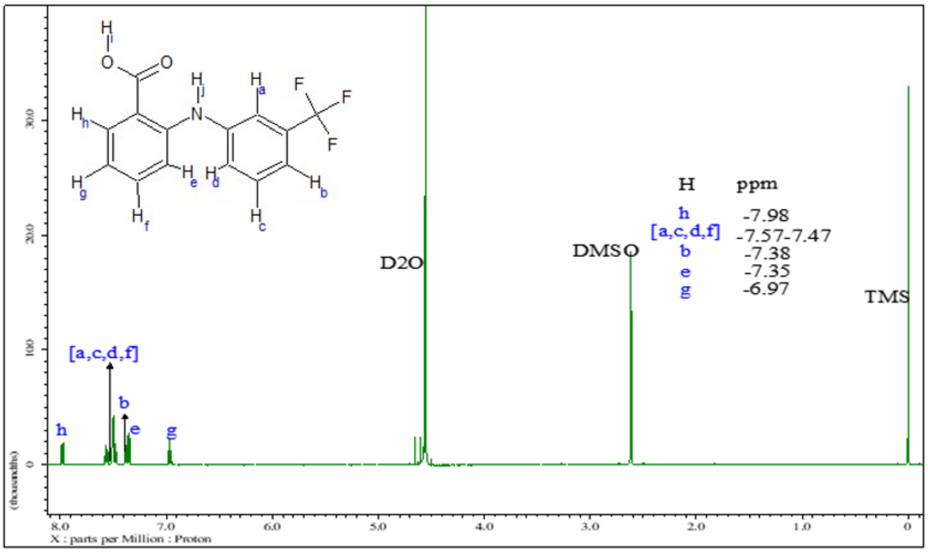

(b) FFA-TP

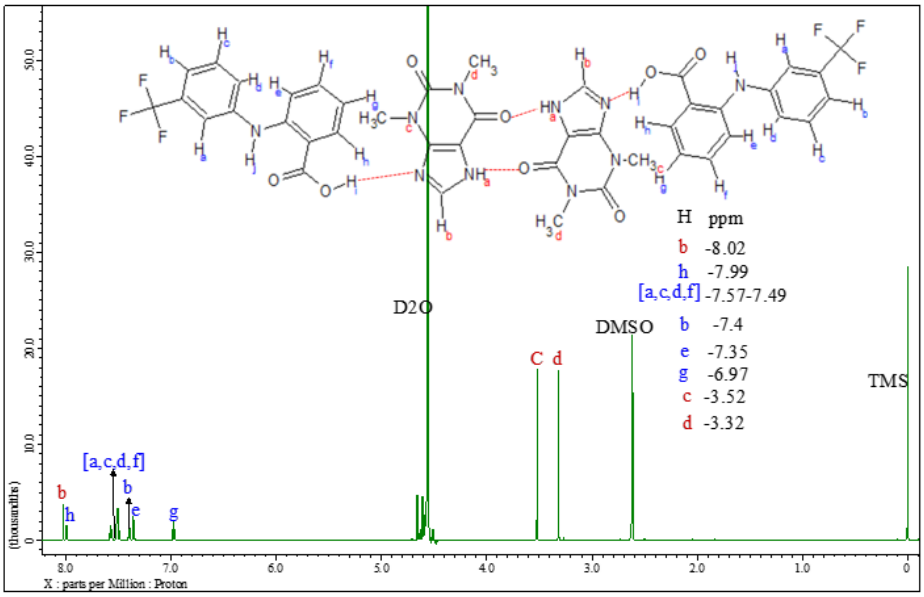

(c) FFA-NIC

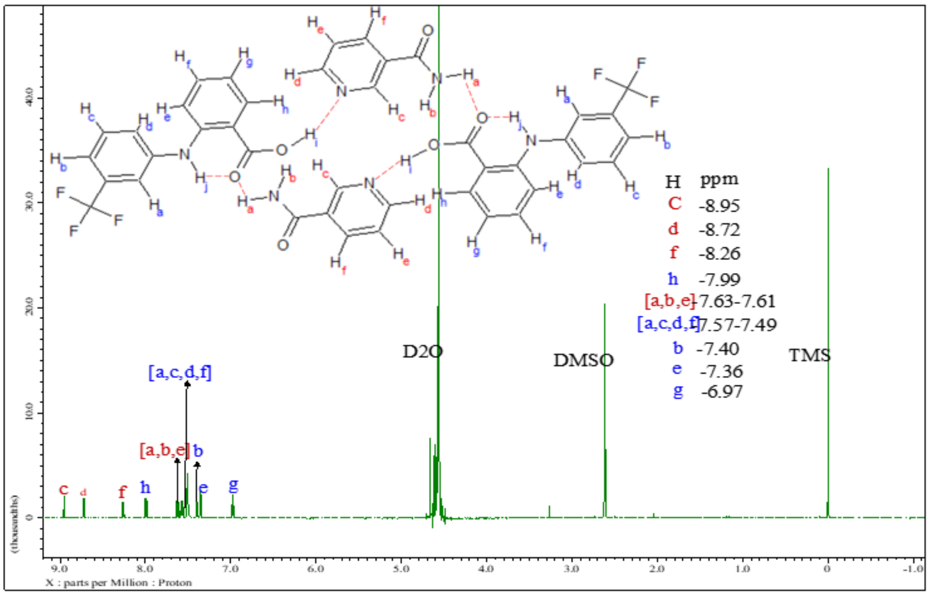

(d) PEG

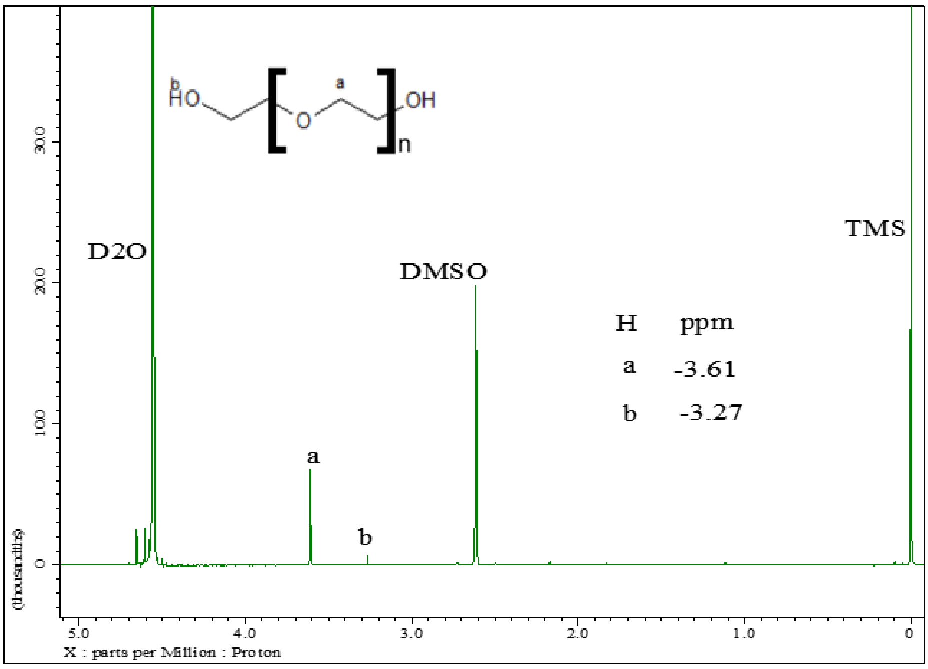

(e) PVPVA

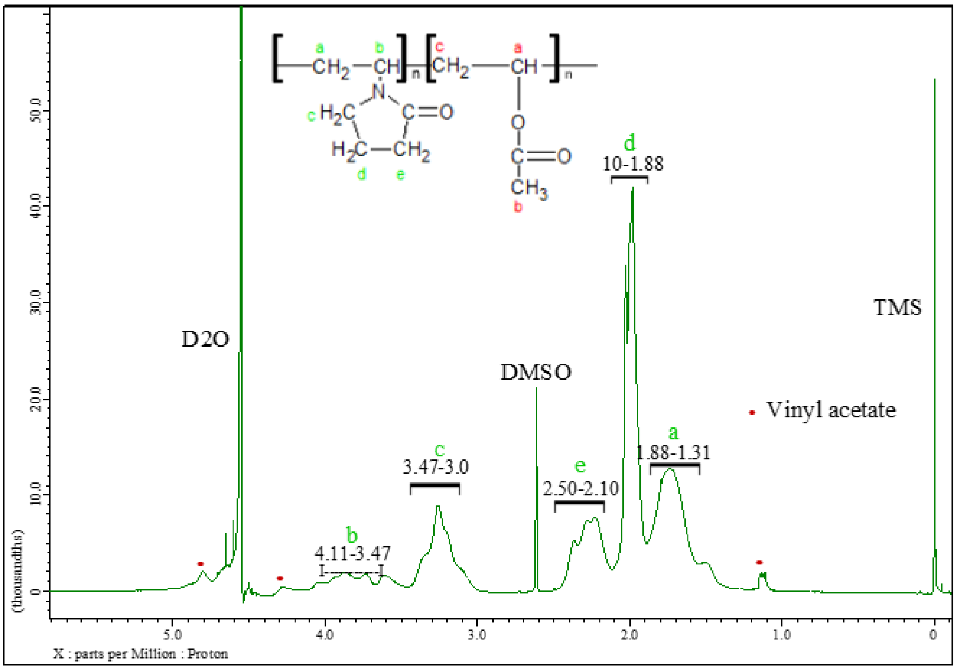

Ref: (PDF) A TG/FTIR study on the thermal degradation of poly(vinyl pyrrolidone) (researchgate.net)

(f) SOL

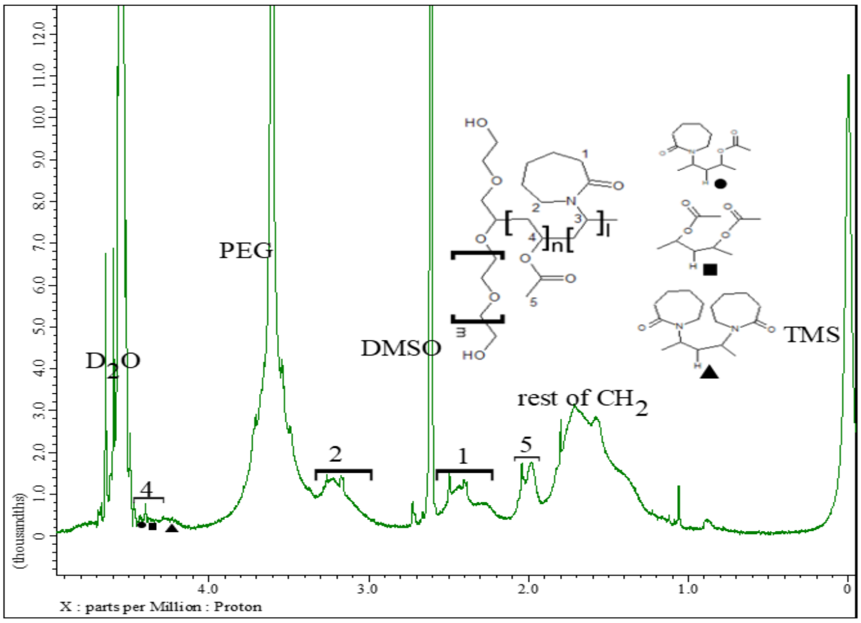

Ref: Self-Assembly of Soluplus in Aqueous Solutions: Characterization and Prospectives on Perfume Encapsulation | ACS Applied Materials & Interfaces

Figure S3: One-dimensional ^{1}H NMR spectra analysis in FFA-TP solution in the absence and presence of a polymer or a combination: (a) PVP-VA; (b) SOL; (c) PEG; (d) SOL & PVP-VA; (e) PVP-VA & PEG

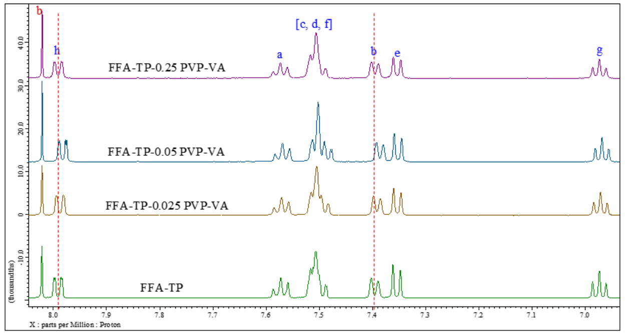

(a) PVP-VA

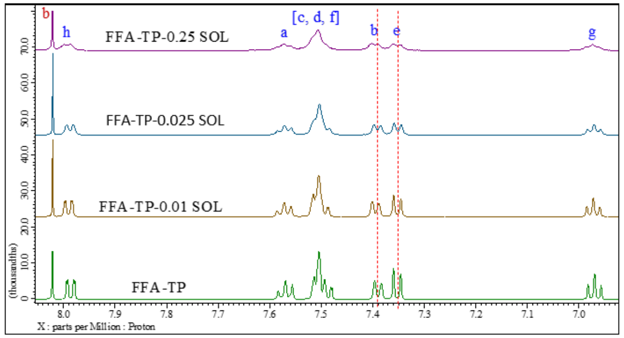

(b) SOL

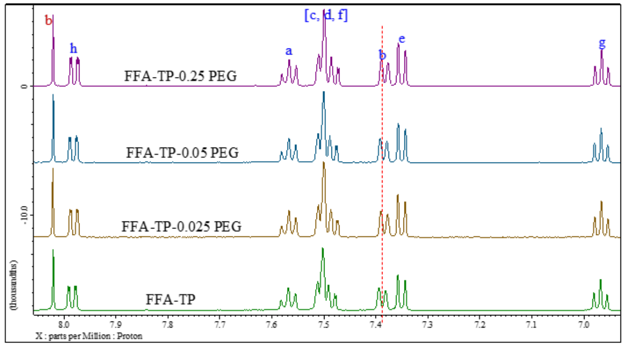

(c) PEG

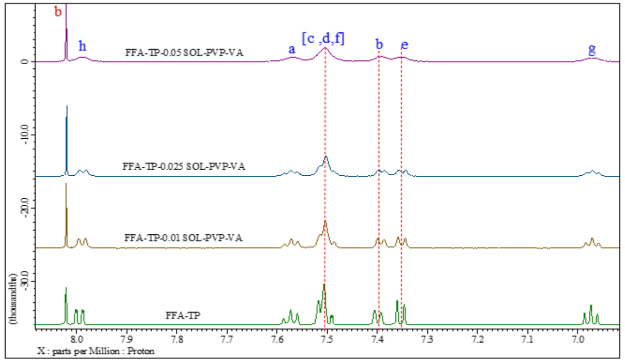

(d) SOL & PVP-VA

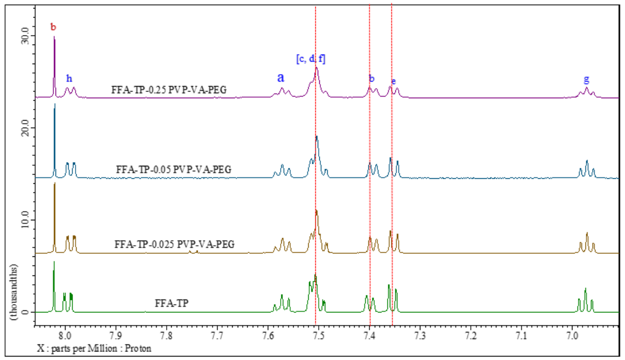

(e) PVP-VA & PEG

Figure S4: One-dimensional ^{1}H NMR spectra analysis in FFA-NIC solution in the absence and presence of a polymer or a combination: (a) PVP-VA; (b) SOL; (c) PEG; (d) SOL & PVP-VA; (e) PVP-VA & PEG

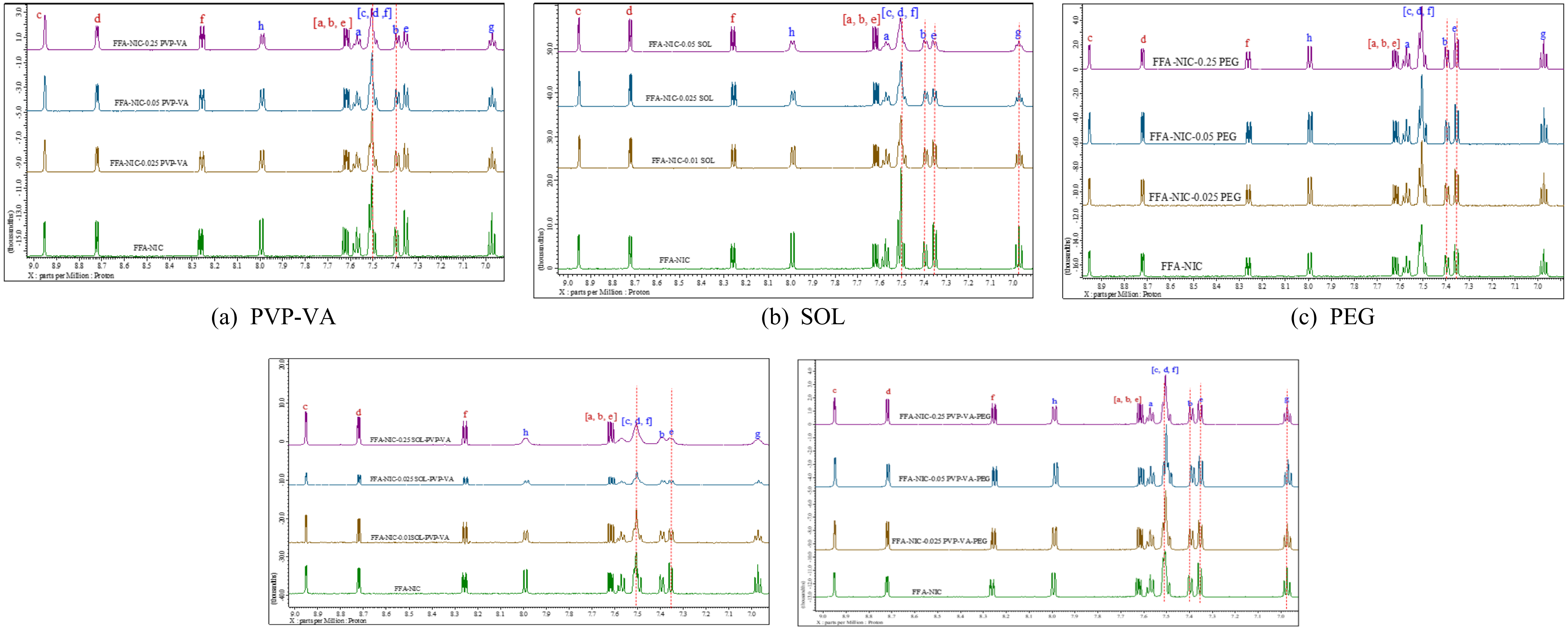

(a) PVP-VA

(b) SOL

(c) PEG

(d) SOL & PVP-VA

(e) PVP-VA & PEG

Figure S5: Comparison of the ^{1}H chemical shifts of the protons of FFA in solution of FFA, FFA-TP, and FFA-NIC in the presence and absence of polymers

	FFA	FFA-TP	FFA-NIC
PVP-VA	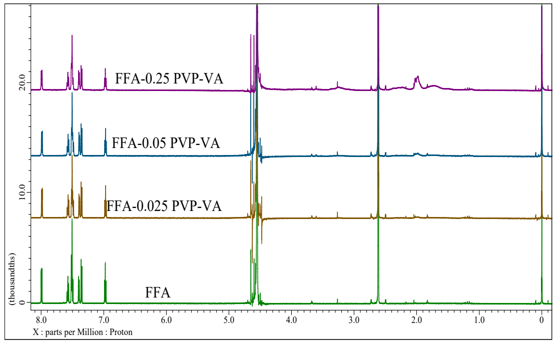	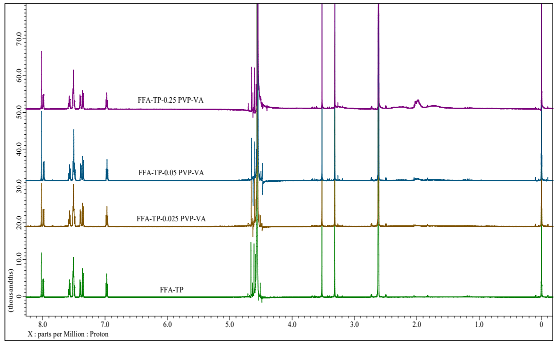	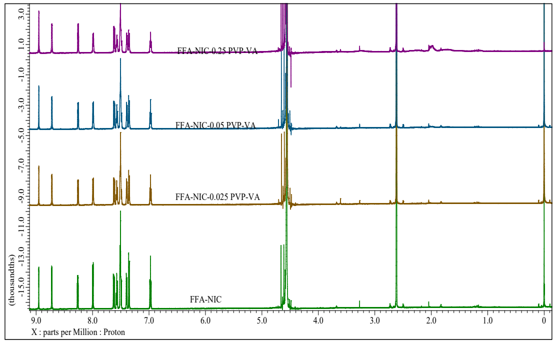
SOL	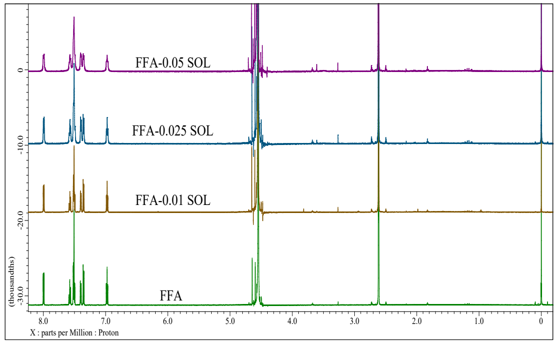	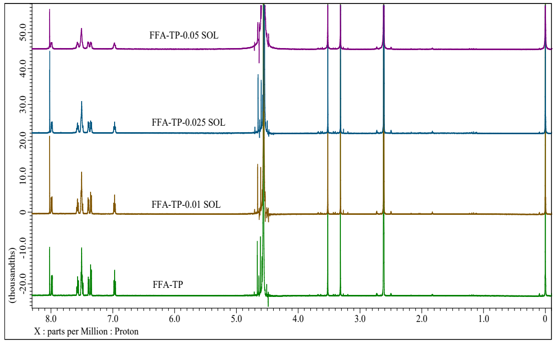	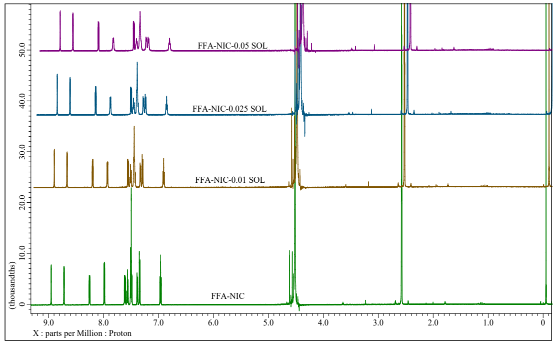

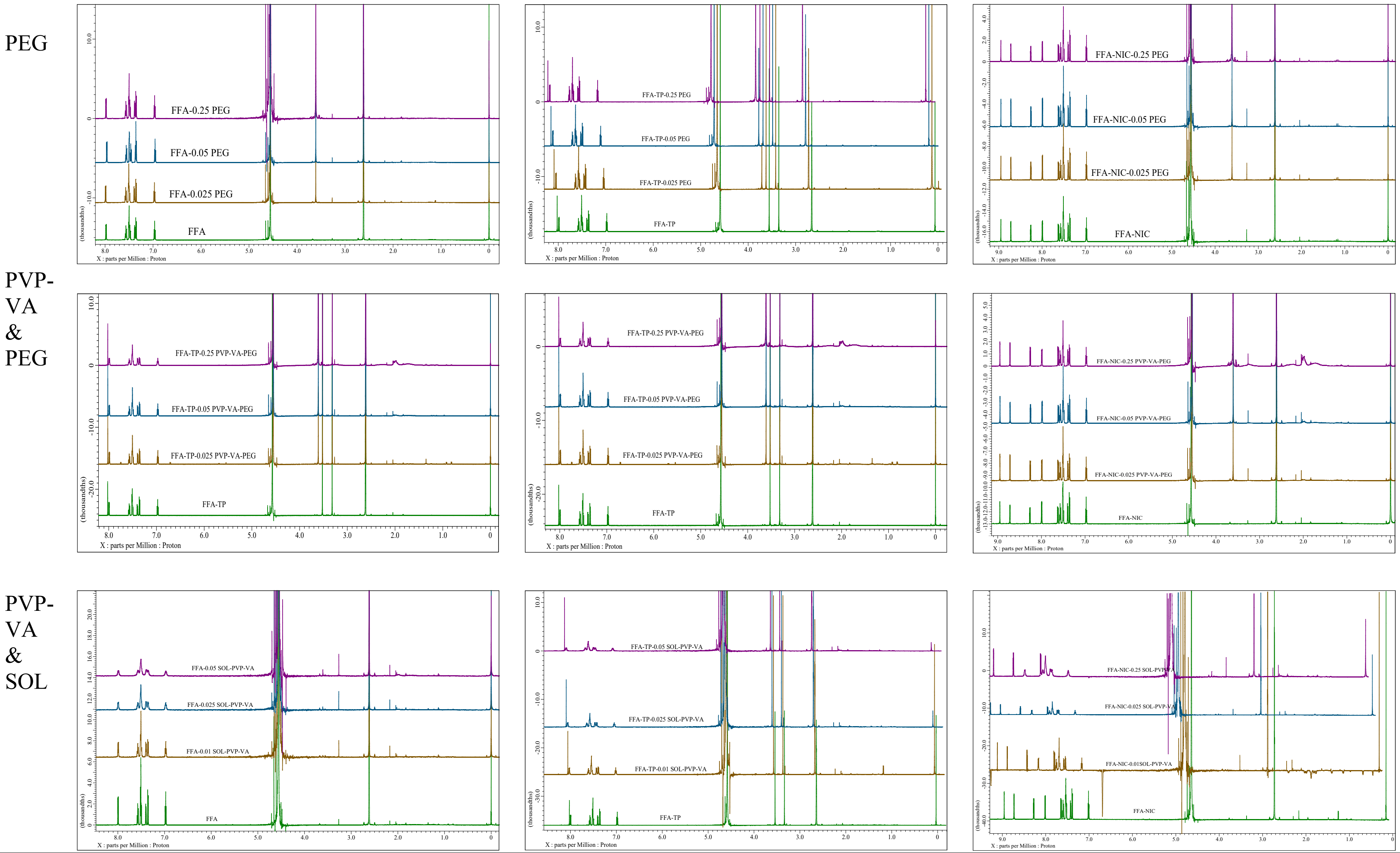
PEG
FFA-0.25 PEG
FFA-0.05 PEG
FFA-0.025 PEG
FFA
FFA-TP-0.25 PEG
FFA-TP-0.05 PEG
FFA-TP-0.025 PEG
FFA-TP
FFA-NIC-0.25 PEG
FFA-NIC-0.05 PEG
FFA-NIC-0.025 PEG
FFA-NIC
PVP-VA & PEG
FFA-TP-0.25 PVP-VA-PEG
FFA-TP-0.05 PVP-VA-PEG
FFA-TP-0.025 PVP-VA-PEG
FFA-TP
FFA-NIC-0.25 PVP-VA-PEG
FFA-NIC-0.05 PVP-VA-PEG
FFA-NIC-0.025 PVP-VA-PEG
FFA-NIC
PVP-VA & SOL
FFA-0.05 SOL-PVP-VA
FFA-0.025 SOL-PVP-VA
FFA-0.01 SOL-PVP-VA
FFA
FFA-TP-0.05 SOL-PVP-VA
FFA-TP-0.025 SOL-PVP-VA
FFA-TP-0.01 SOL-PVP-VA
FFA-TP
FFA-NIC-0.25 SOL-PVP-VA
FFA-NIC-0.025 SOL-PVP-VA
FFA-NIC-0.01SOL-PVP-VA
FFA-NIC
X : parts per Million : Proton
(thousandths)

Figure S6: NOESY spectral of solutions of FFA, FFA-TP, and FFA-NIC in the presence and absence of polymer in DMS06-D20 solvent

Polymer	FFA	FFA-TP	FFA-NIC

0.025 mg/mL

PVP-VA

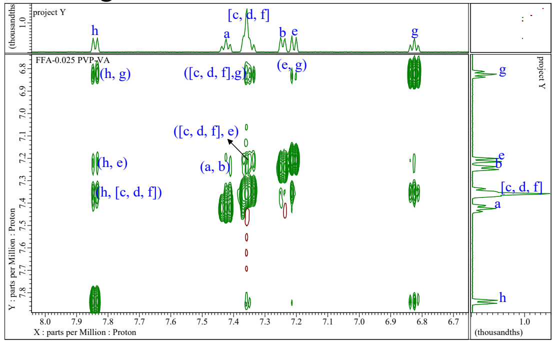

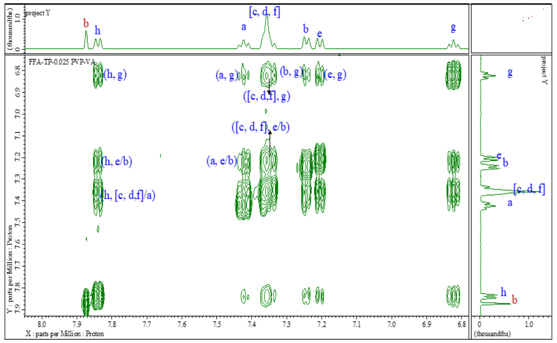

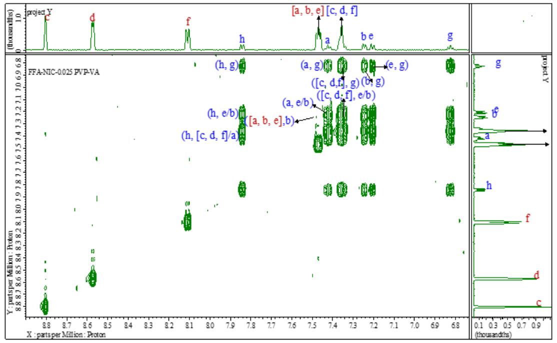

0.05 mg/mL

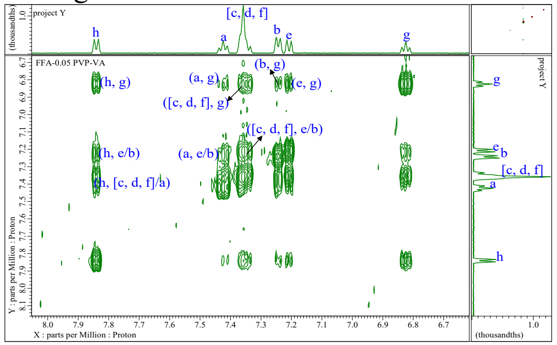

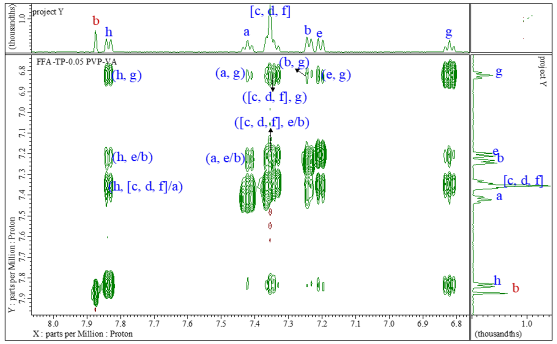

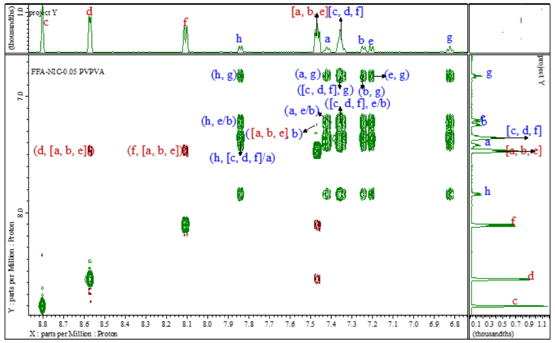

0.25 mg/mL

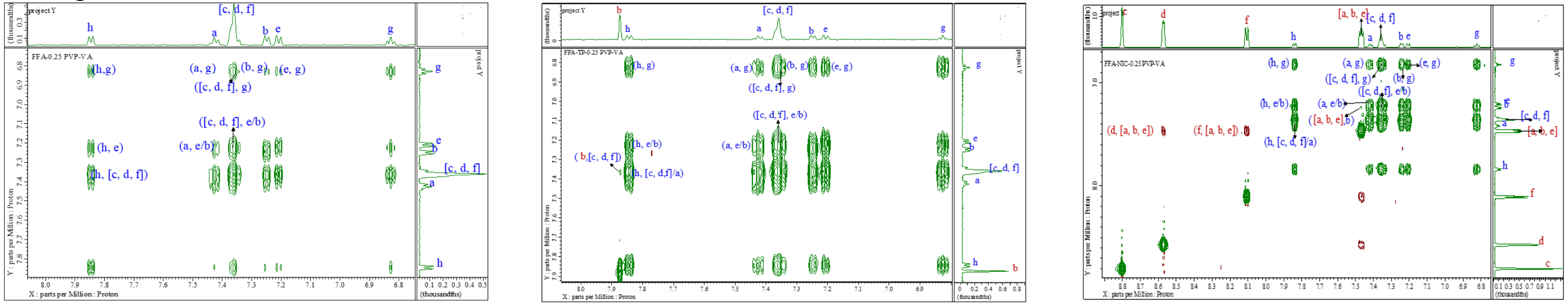

SOL

0.01 mg/mL

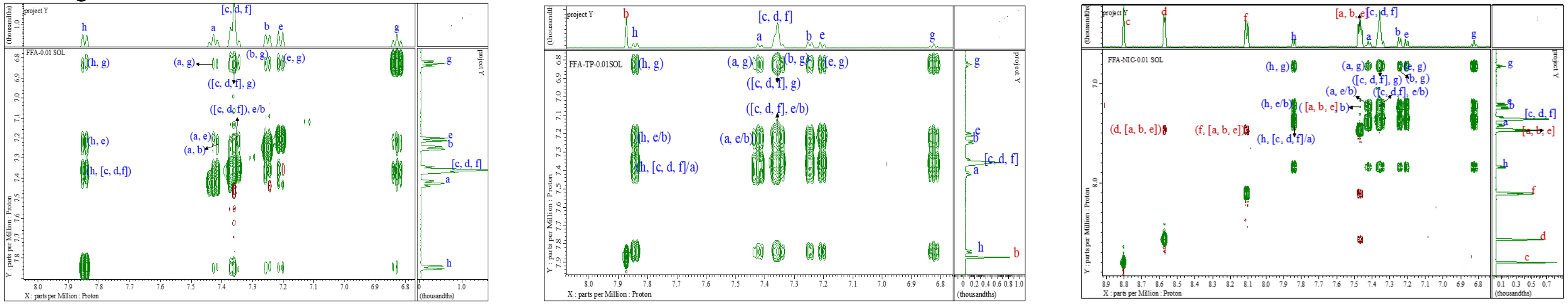

0.025 mg/mL

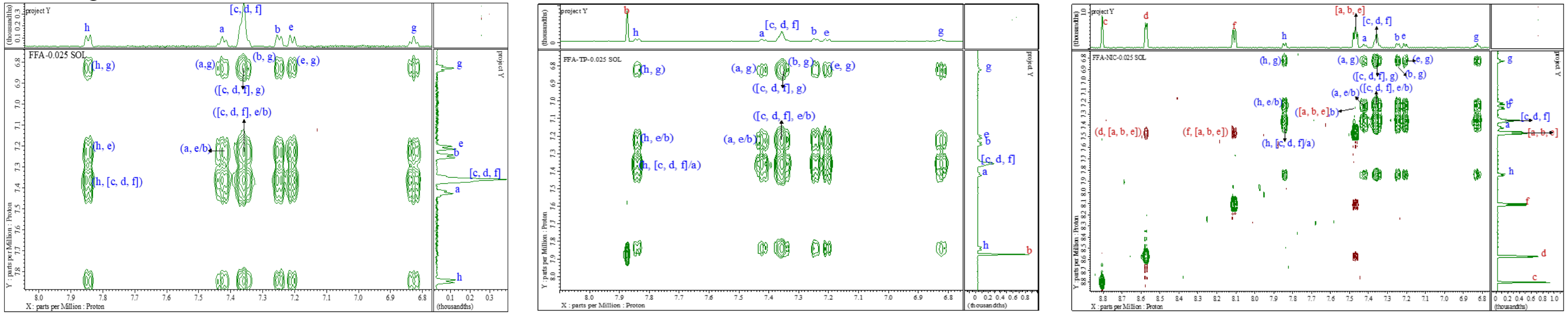

0.05 mg/mL

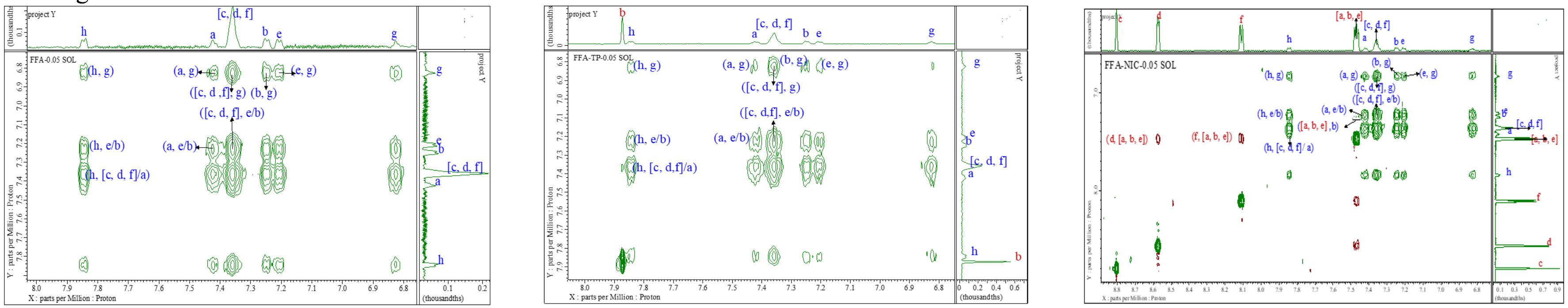

PEG

0.025 mg/mL

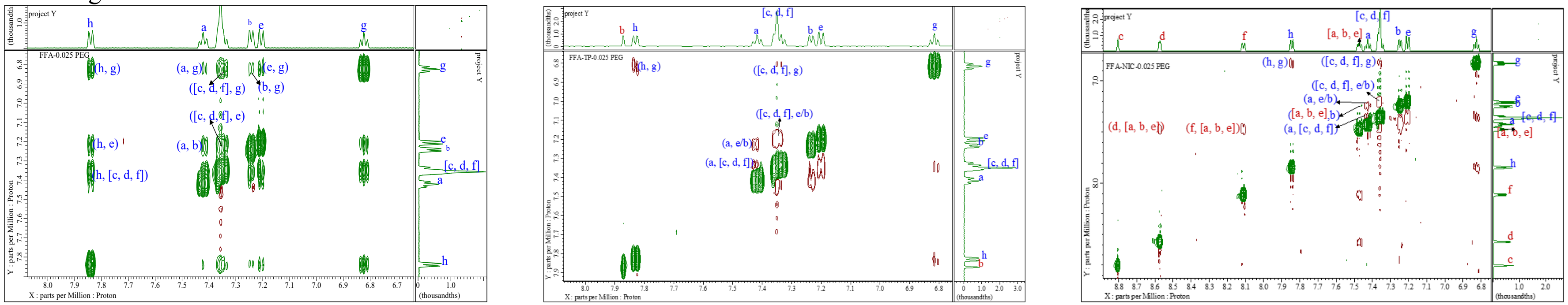

0.05 mg/mL

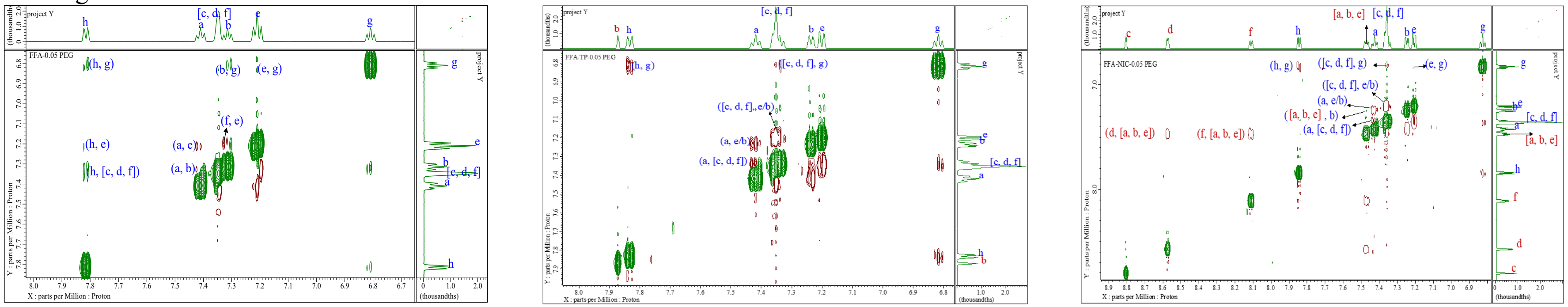

0.25 mg/mL

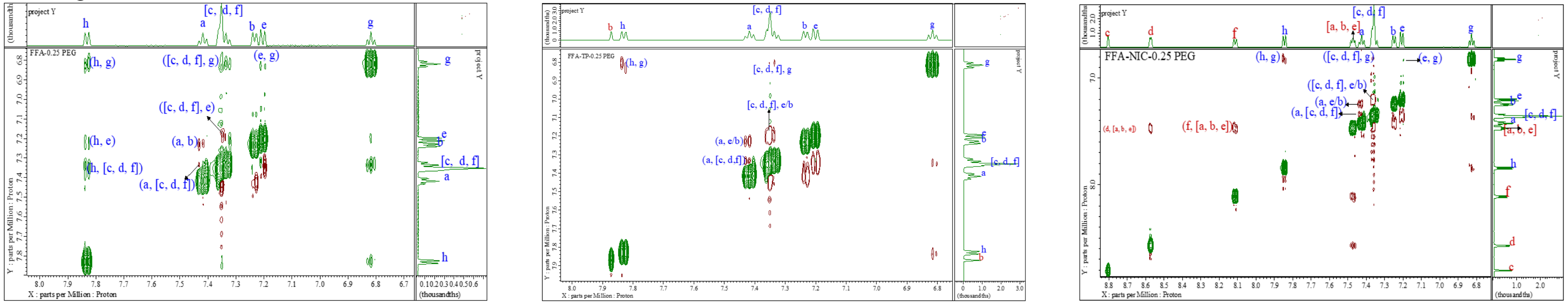

PVP-VA & PEG

0.025 mg/mL

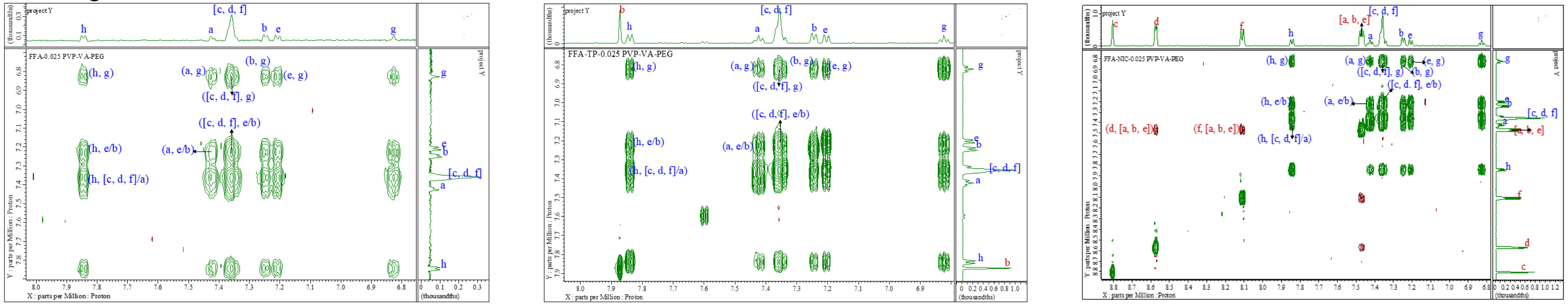

0.05 mg/mL

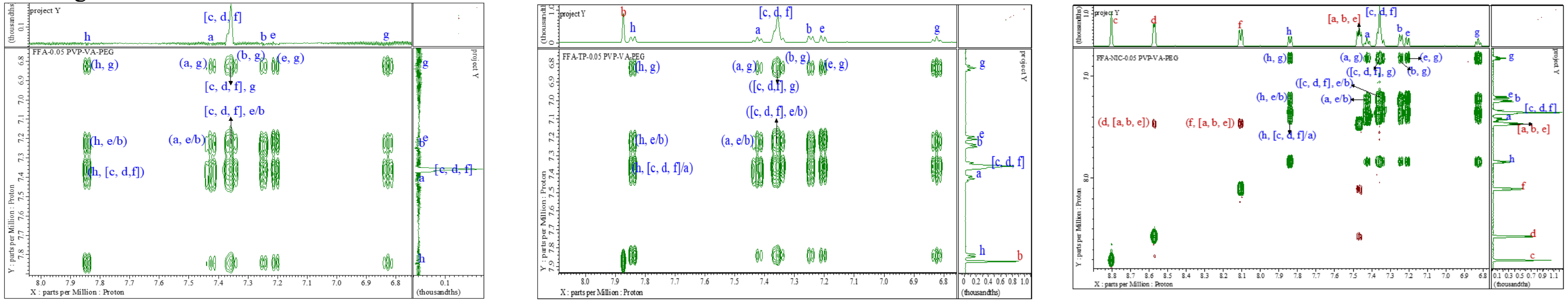

0.25mg/mL

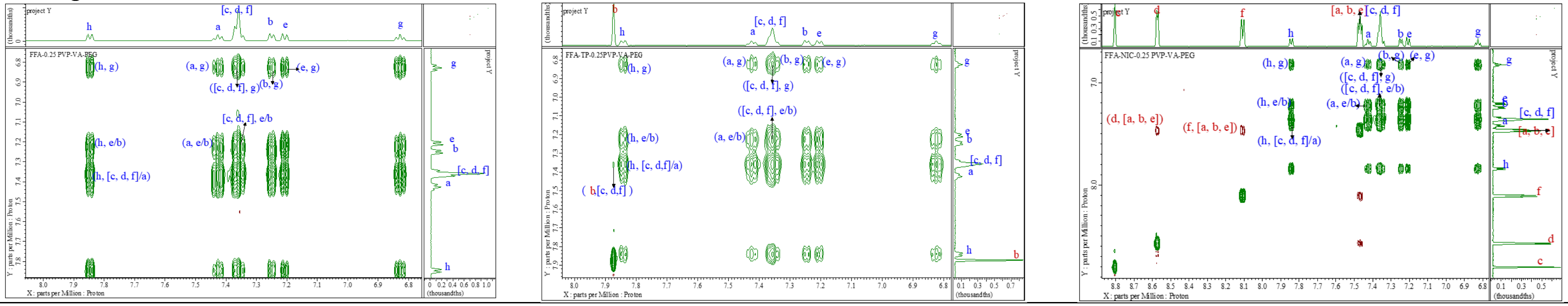

PVP-VA & SOL

0.01 mg/mL

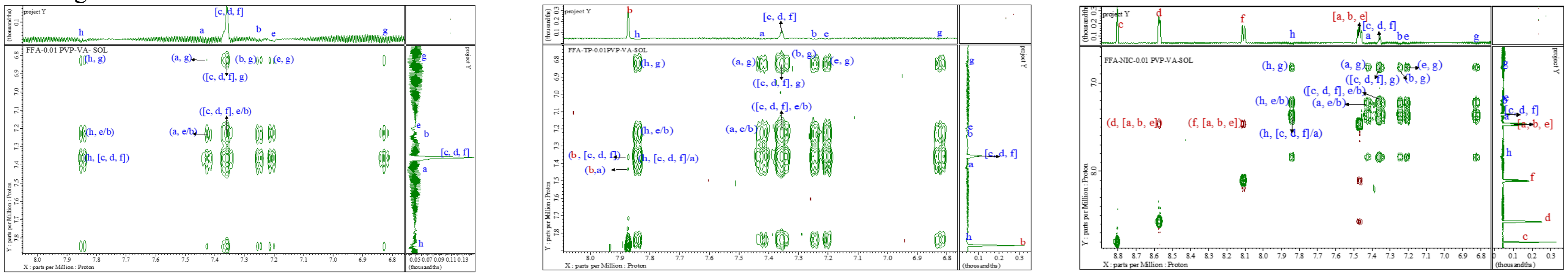

0.025 mg/mL

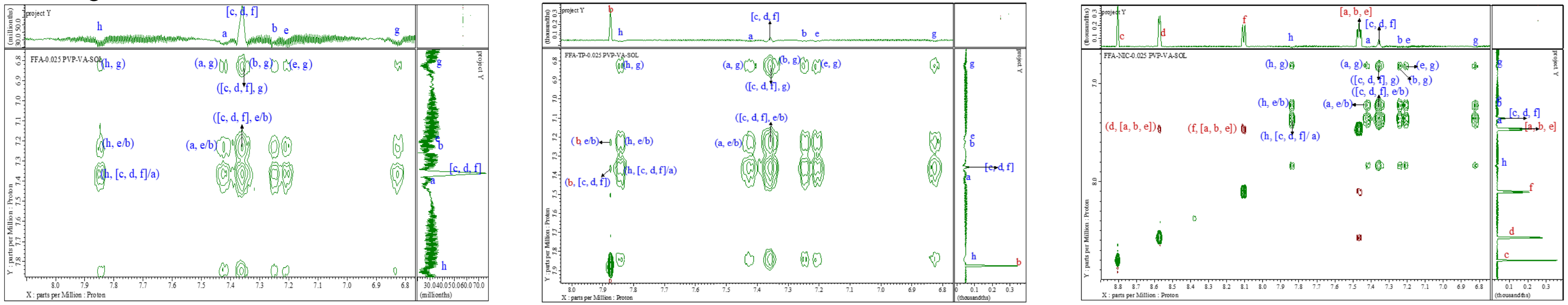

0.05 mg/mL

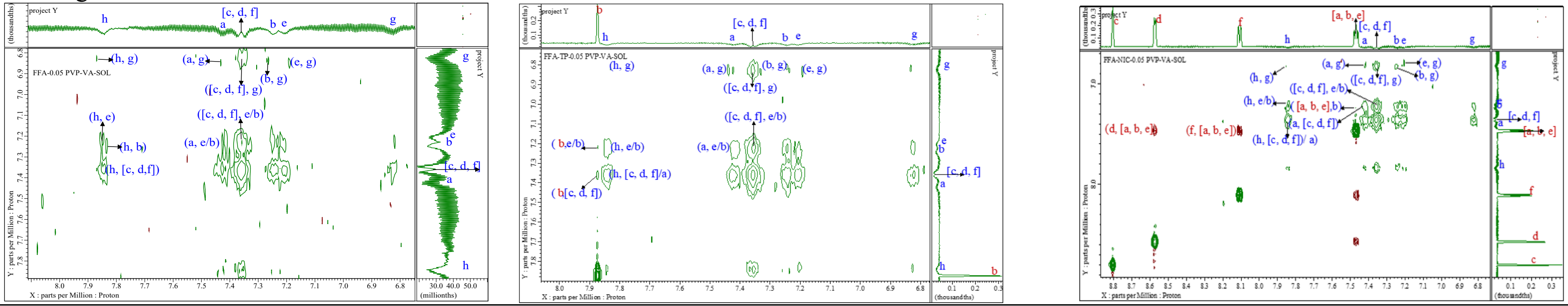

Figure S7: DOSY spectral of FFA solution with polymer(s) in DMS06-D20 solvent

	FFA	FFA-TP	FFA-NIC
NO POLYMER	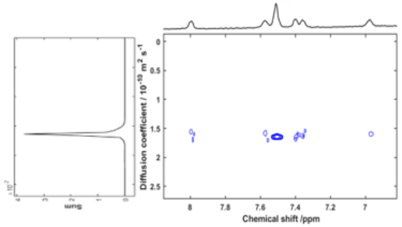	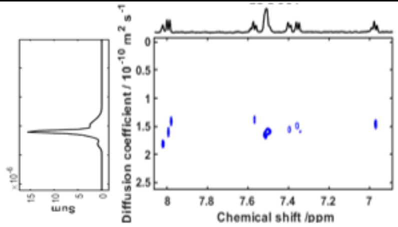	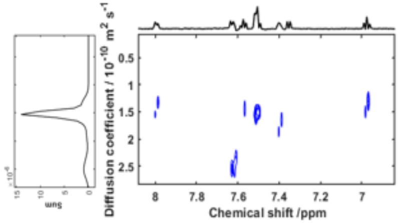
PVPVA 0.025	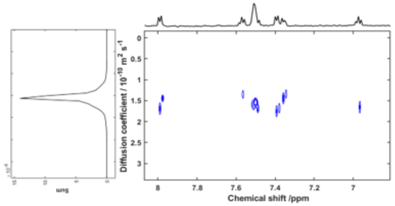	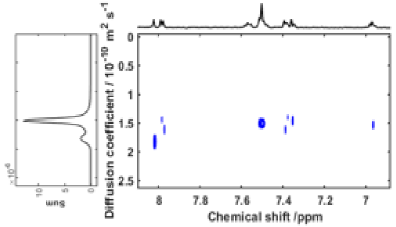	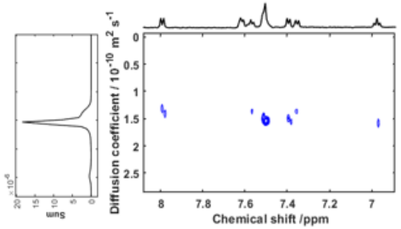
0.05	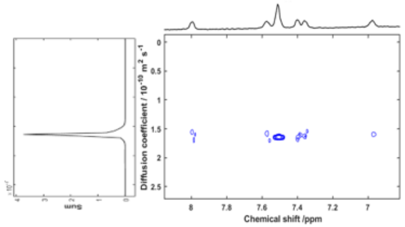	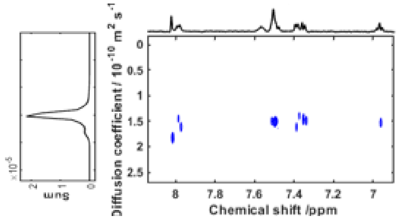	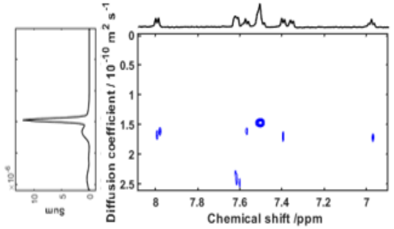
0.25	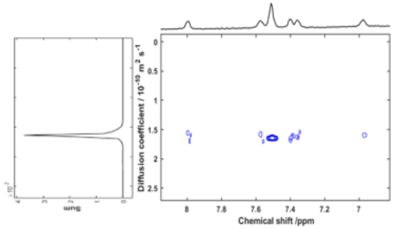	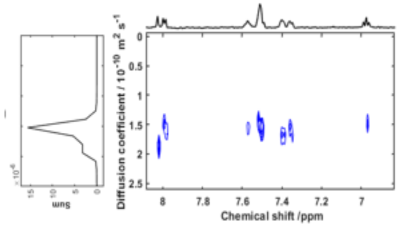	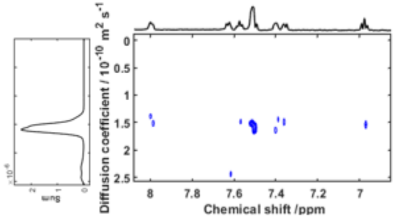

PEG

0.025

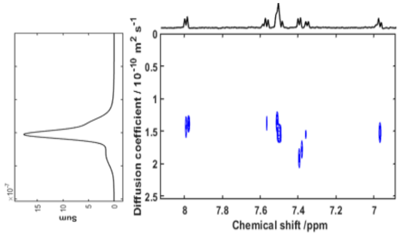

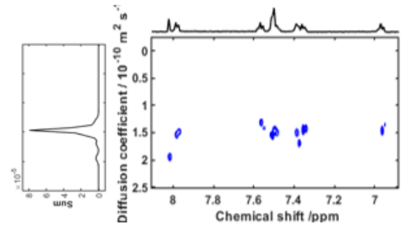

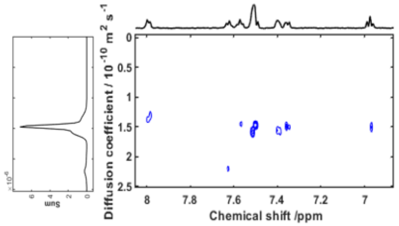

0.05

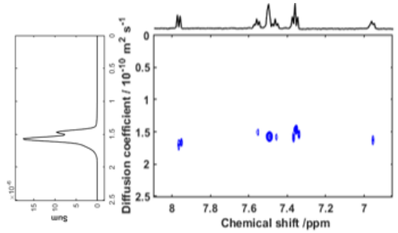

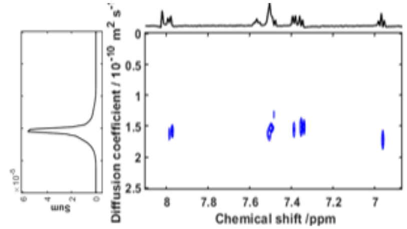

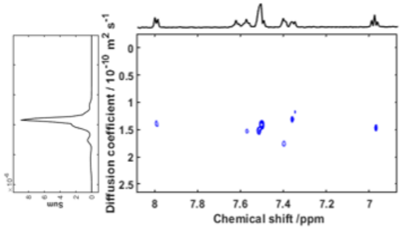

0.25

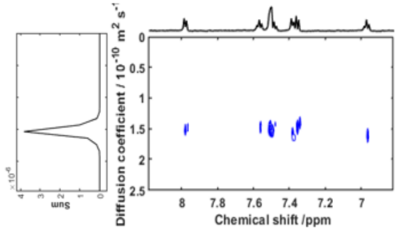

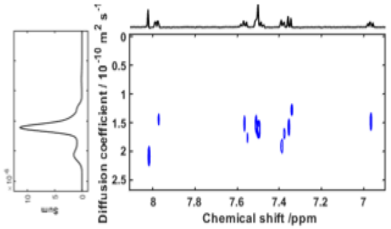

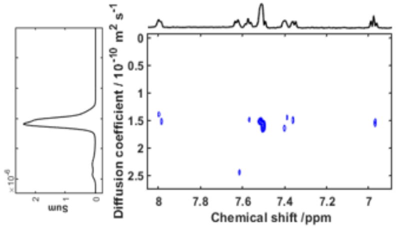

SOL

0.01

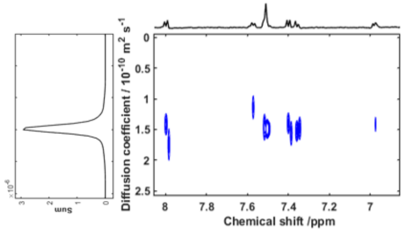

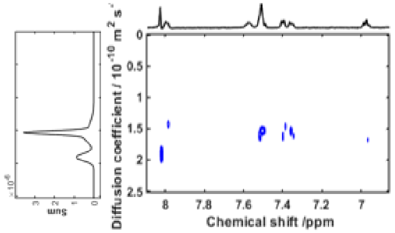

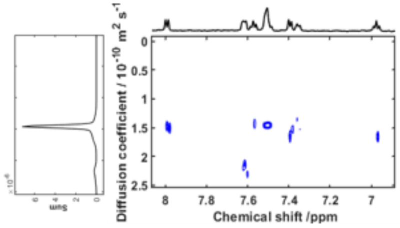

0.025

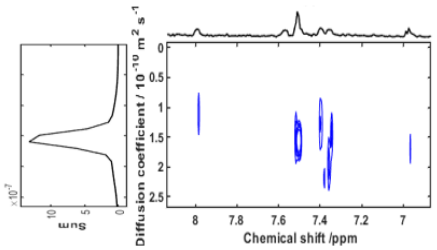

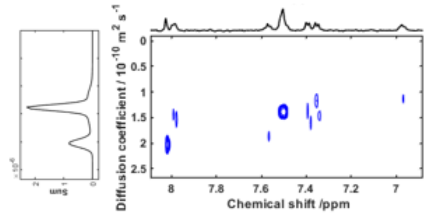

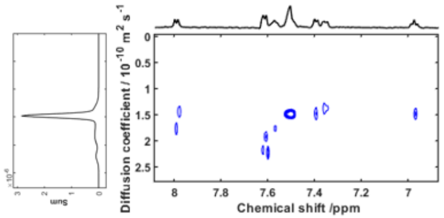

0.05

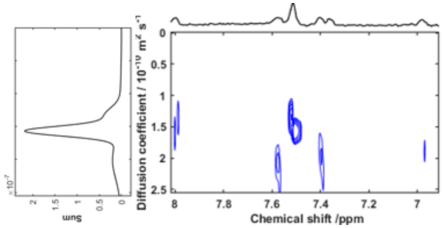

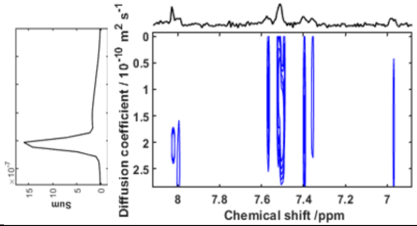

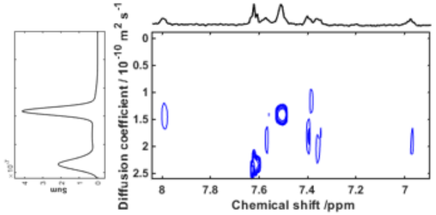

PVP-VA & PEG

0.025

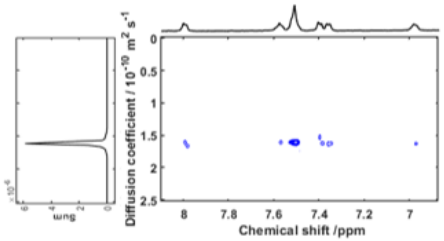

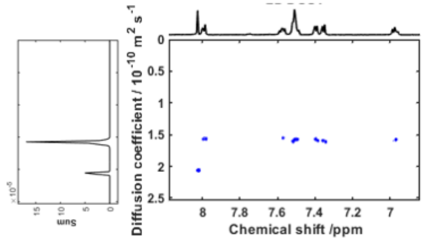

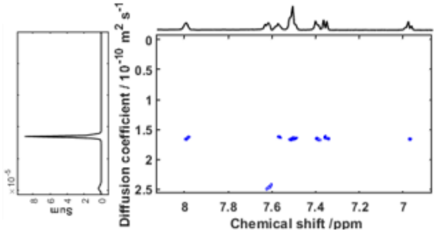

0.05

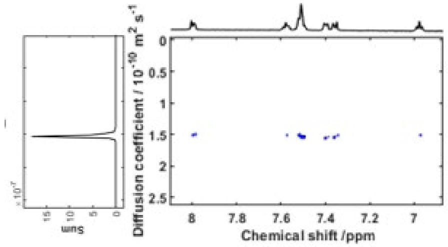

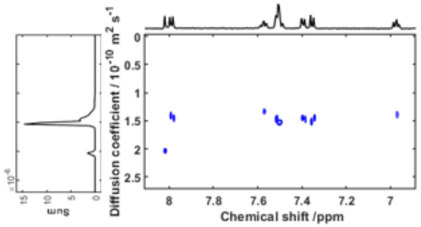

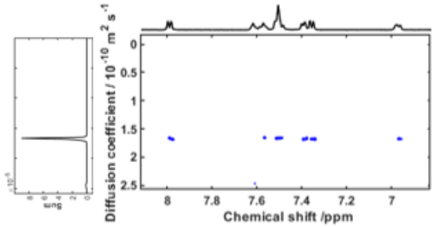

0.25

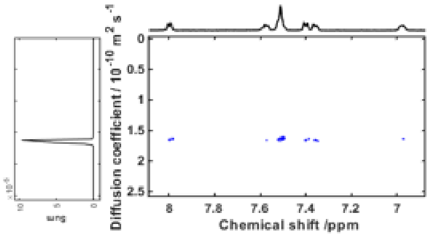

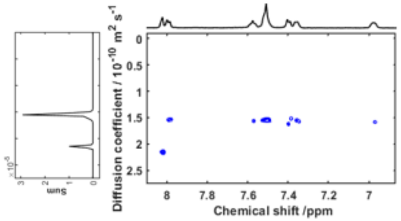

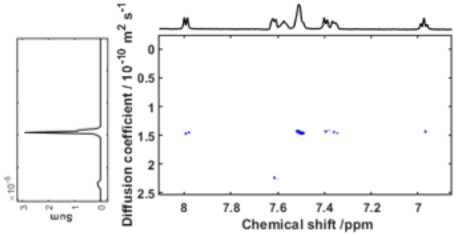

PVP-VA & SOL

0.01

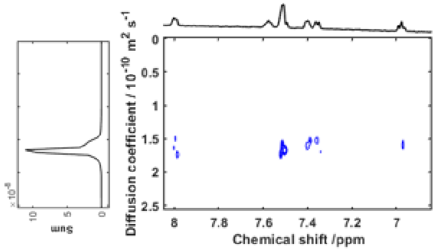

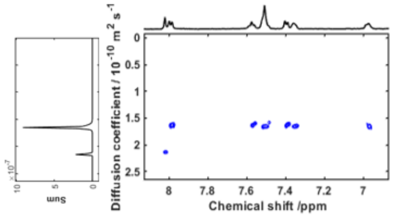

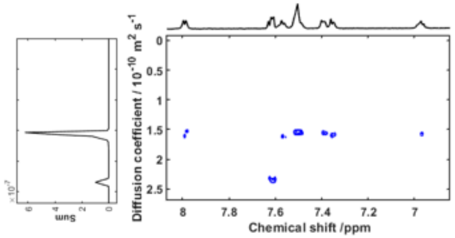

0.025

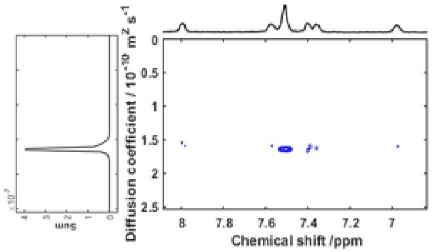

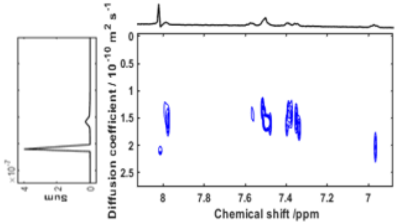

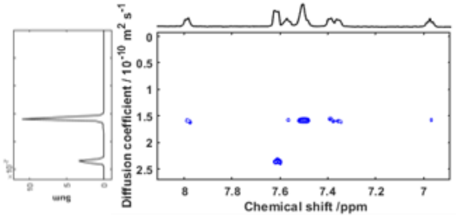

0.05

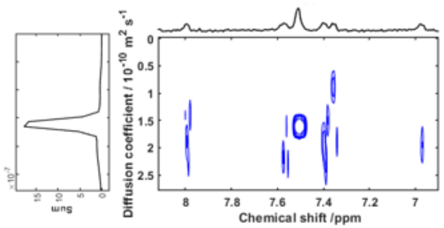

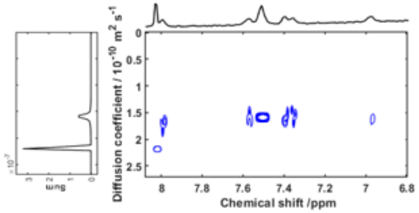

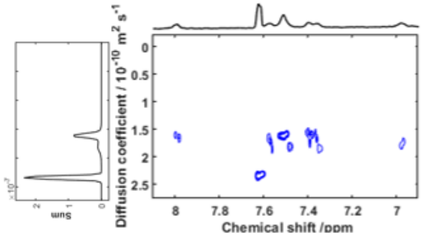

Figure S8: Initial distances between molecules of FFA, TP, NIC and the polymers in the simulations.

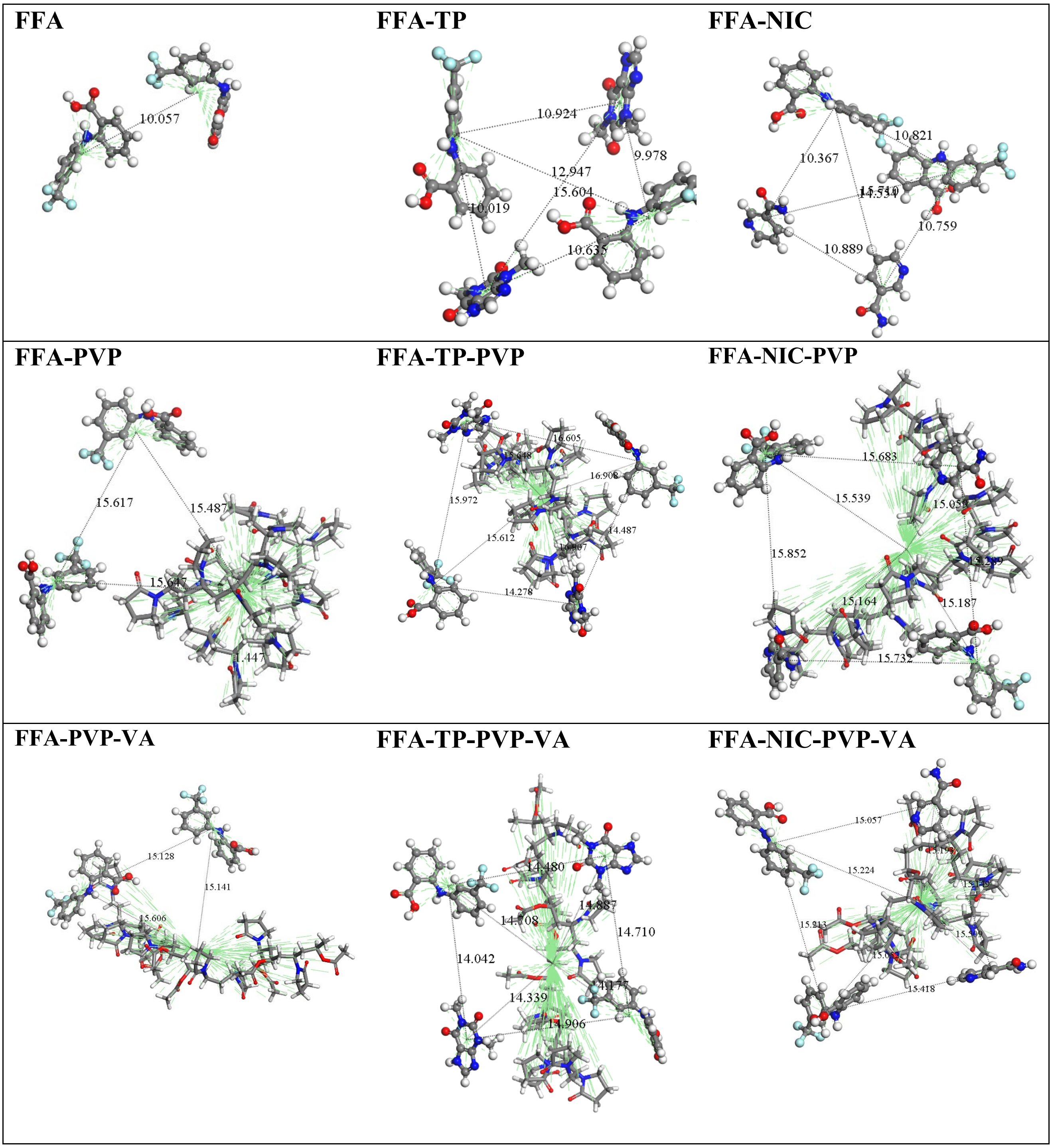

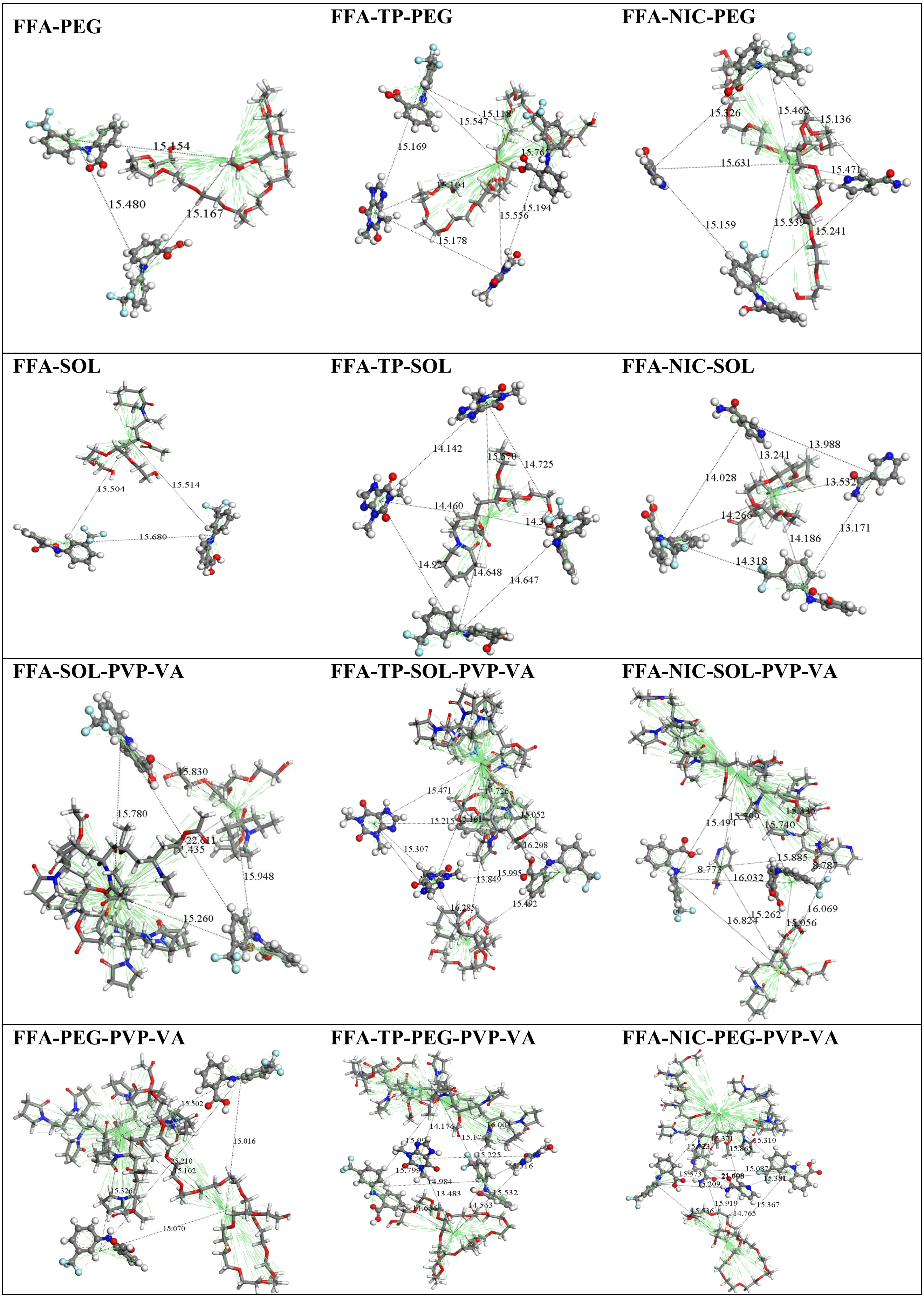
FFA-PEG
FFA-TP-PEG
FFA-NIC-PEG
FFA-SOL
FFA-TP-SOL
FFA-NIC-SOL
FFA-SOL-PVP-VA
FFA-TP-SOL-PVP-VA
FFA-NIC-SOL-PVP-VA
FFA-PEG-PVP-VA
FFA-TP-PEG-PVP-VA
FFA-NIC-PEG-PVP-VA

Figure S9: Distance evolution between the FFA molecules in the FFA, FFA-TP and FFA-NIC simulations.

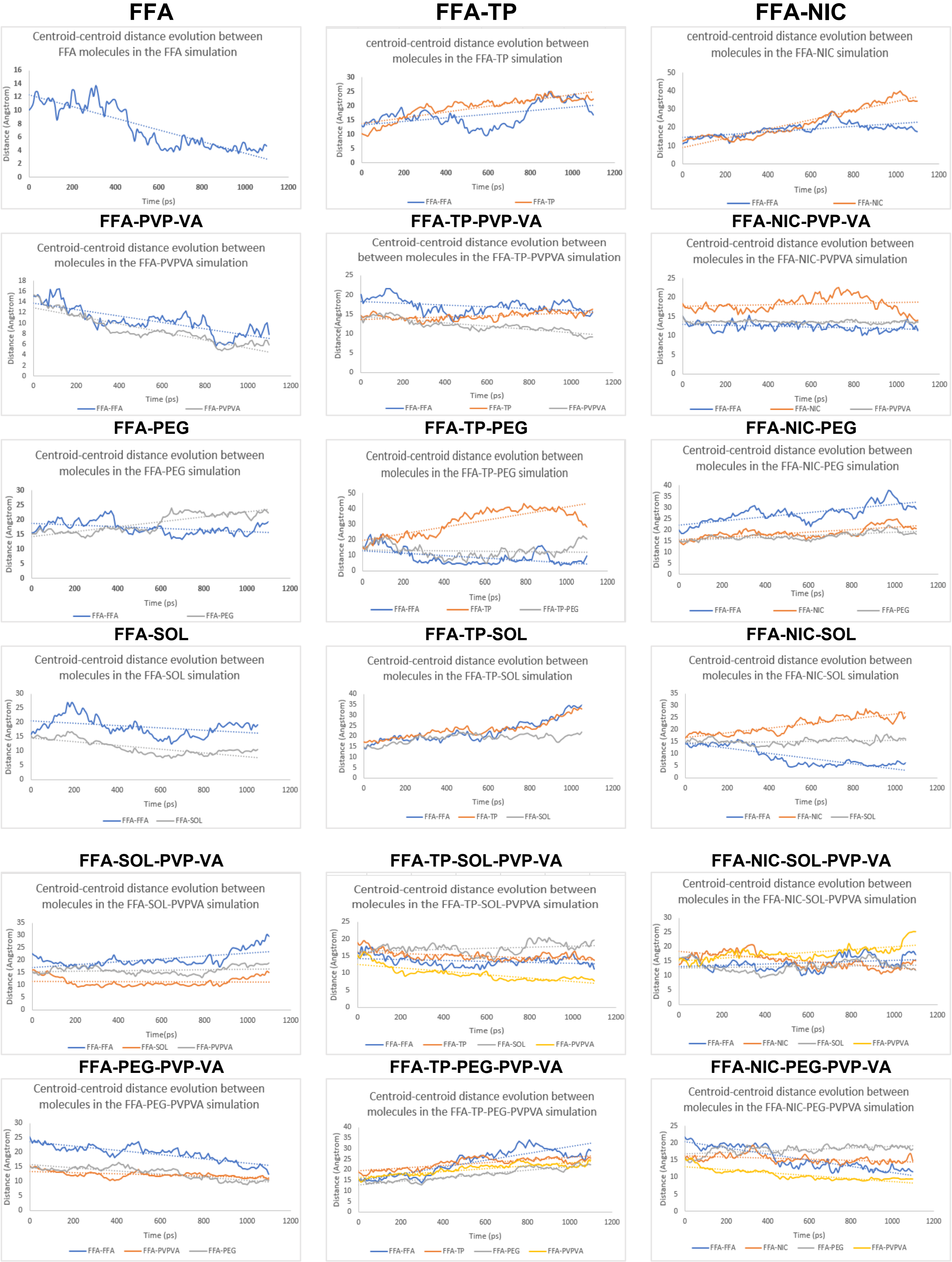

Figure S10: Mean square displacement (MSD) and diffusion coefficient of FFA molecules

<table>
<tr><th>Simulation</th><th>MSD</th><th>Diffusion coefficient of FFA (m^2s^{-1})</th><th>∆ Diffusion coefficient (m^2s^{-1})</th></tr>
<tr><td>FFA</td><td>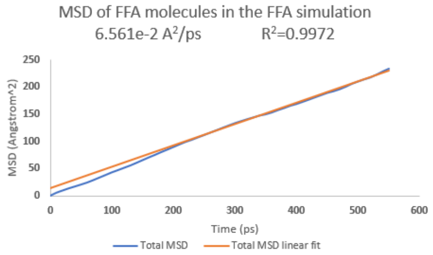
</td><td>6.561 x 10^{-10}</td><td>-</td></tr>
<tr><td>FFA-PVP-VA</td><td>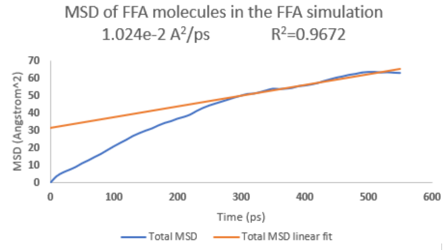
</td><td>1.024 x 10^{-10}</td><td>-5.54E^{-10}</td></tr>
<tr><td>FFA-PEG</td><td>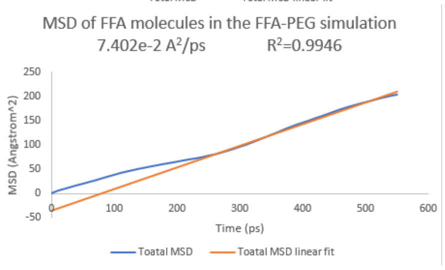
</td><td>7.402 x 10^{-10}</td><td>8.41E^{-11}</td></tr>
<tr><td>FFA-SOL</td><td>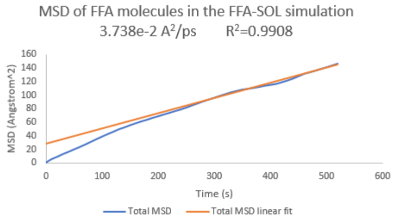
</td><td>3.738 X 10^{-10}</td><td>-2.82E^{-10}</td></tr>
<tr><td>FFA-SOL-PVP-VA</td><td>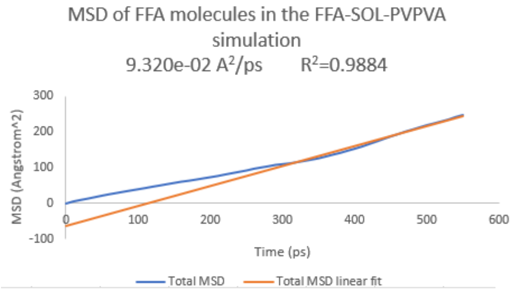
</td><td>9.320 X 10^{-10}</td><td>2.76E^{-10}</td></tr>
<tr><td>FFA-PEG-PVP-VA</td><td>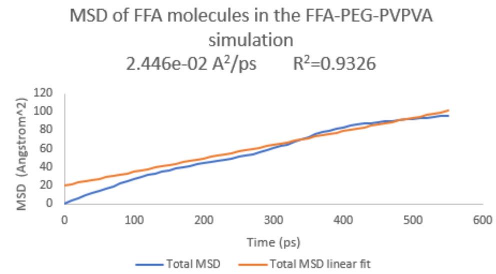
</td><td>2.446 X 10^{-10}</td><td>-4.12E^{-10}</td></tr>
</table>

FFA-TP	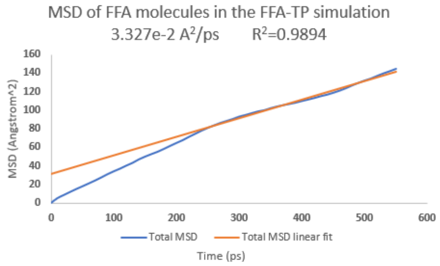	3.327 x 10^{-10}	-
FFA-TP-PVP-VA	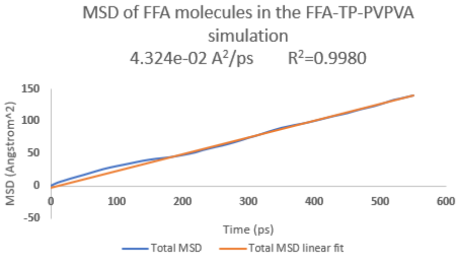	4.324 x 10^{-10}	$9.97E^{-11}$
FFA-TP-PEG	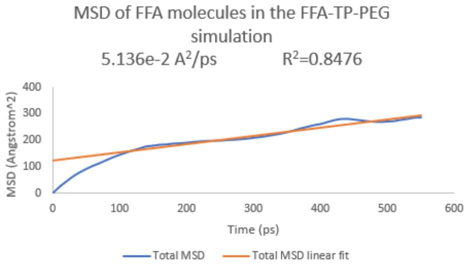	5.136 X 10^{-10}	$1.81E^{-10}$
FFA-TP-SOL	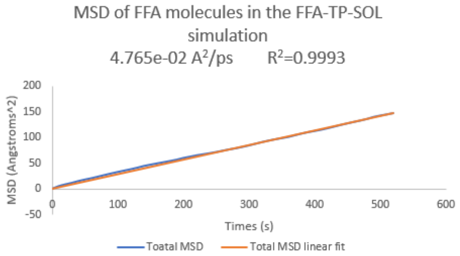	4.765 X 10^{-10}	$1.44E^{-10}$
FFA-TP-SOL-PVP-VA	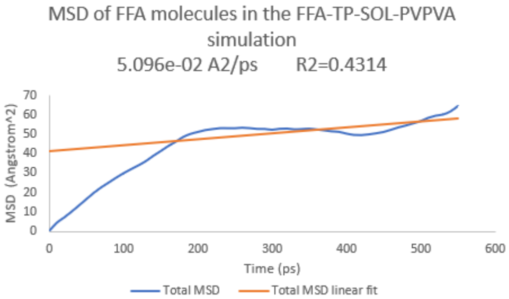	5.096 X 10^{-10}	$1.77E^{-10}$
FFA-TP-PEG-PVP-VA	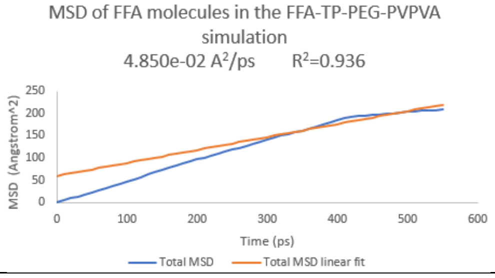	4.850 X 10^{-10}	$1.52E^{-10}$

FFA-NIC	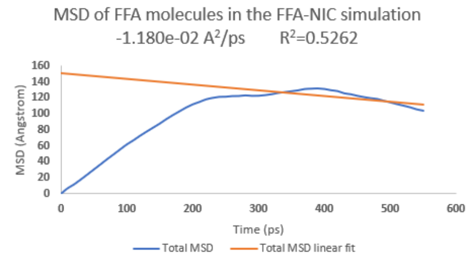	-1.180 x 10^{-10}	-
FFA-NIC-PVP-VA	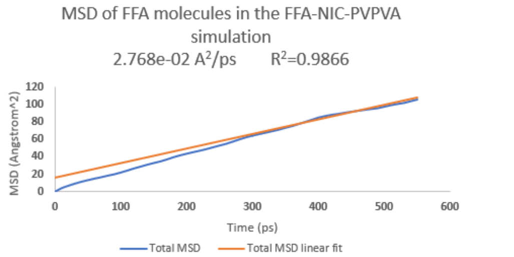	2.768 x 10^{-10}	3.95E^{-10}
FFA-NIC-PEG	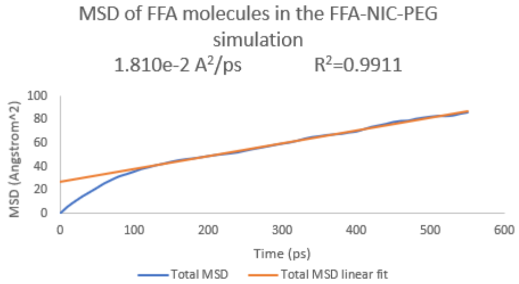	1.810 X 10^{-10}	2.99E^{-10}
FFA-NIC-SOL	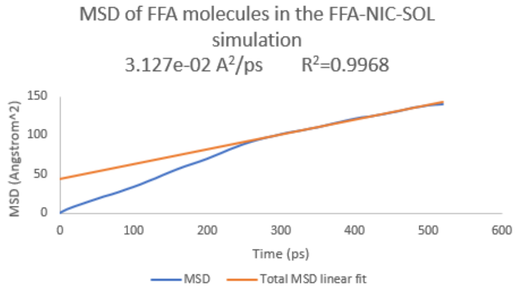	3.127 X 10^{-10}	4.31E^{-10}
FFA-NIC-SOL-PVPVA	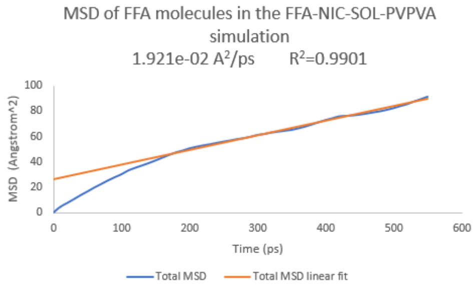	1.921 X 10^{-10}	3.10E^{-10}
FFA-NIC-PEG-PVP-VA	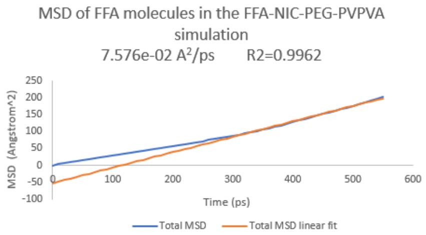	7.576 X 10^{-10}	8.76E^{-10}

Figure S11: Radial Distribution Function (RDF) of the molecules in the FFA, FFA-TP and FFA-NIC simulation

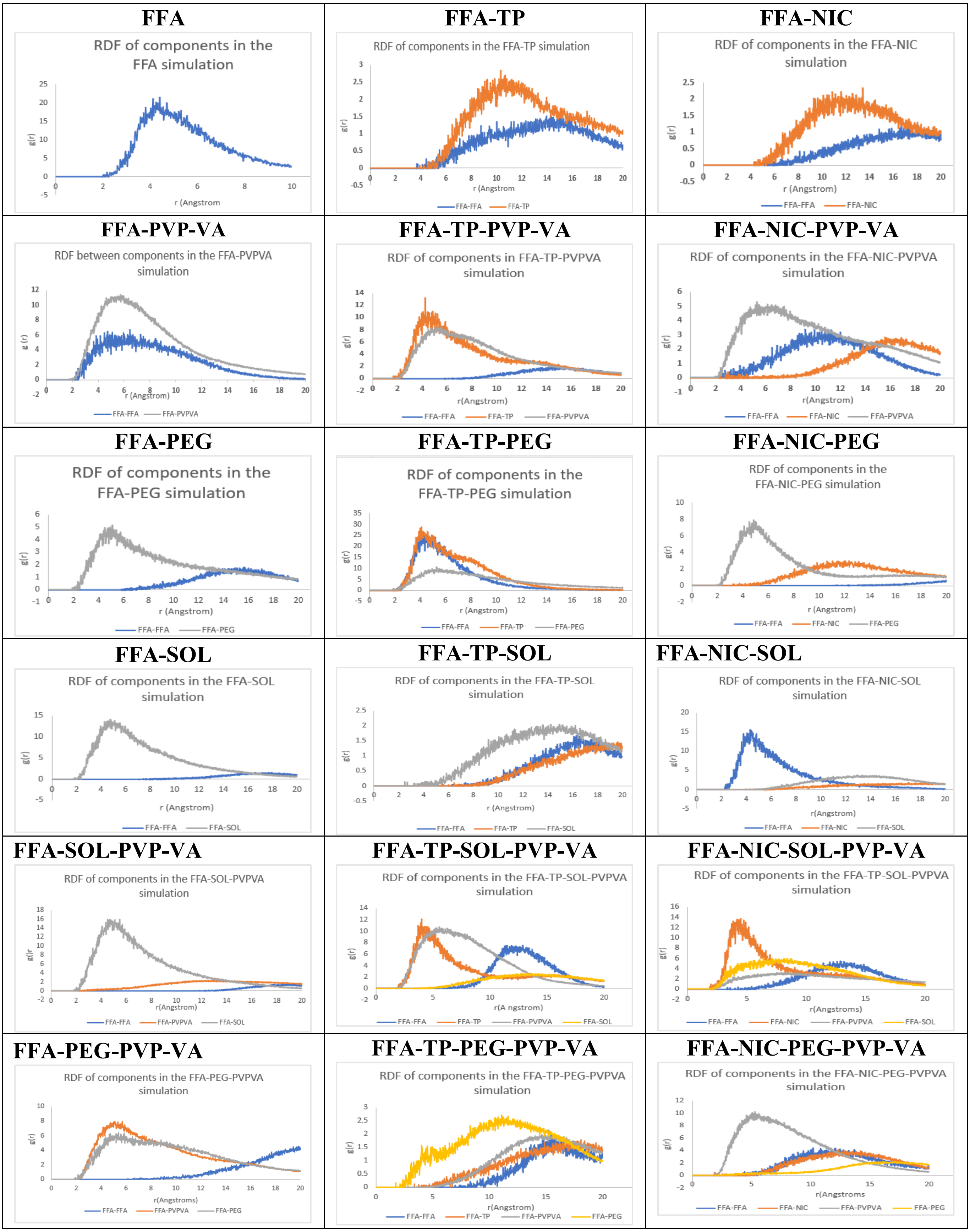

Supplement: Supplementary file 1 — cg4c01573_si_001.pdf [file cg4c01573_si_001.pdf]
